# Research prioritisation in preparedness for and response to outbreaks of high-consequence pathogens: a scoping review

**DOI:** 10.1186/s12916-025-03973-8

**Published:** 2025-03-10

**Authors:** Emilia Antonio, Nicolas Pulik, Susan Khader Ibrahim, Adebisi Adenipekun, Shanthi Levanita, Isabel Foster, Dorothy Chepkirui, Eli Harriss, Louise Sigfrid, Alice Norton

**Affiliations:** 1 Policy and Practice Research Group, Pandemic Sciences Institute, University of Oxford, Oxford, OX3 7DQ UK; 2 Bodleian Health Care Libraries, University of Oxford, Oxford, OX3 9DU UK

**Keywords:** Research prioritisation, Outbreaks, High-consequence pathogens, Response, Preparedness

## Abstract

**Background:**

Priority setting for research on epidemic/pandemic-prone pathogens is essential for the allocation of limited resources to optimise impact. It involves the identification of gaps in knowledge crucial to effective preparedness and response to outbreaks. This review maps priority-setting exercises, reviews their approaches to research prioritisation and describes associated monitoring and evaluation processes for research priorities on high-consequence pathogens.

**Methods:**

Using search terms associated with high-consequence pathogens, as defined by the WHO (2020), EMERGE (2019), European CDC (2022) and the Association of Southeast Asian Nations (2021), and research prioritisation, we searched WHO Global Index Medicus; Ovid Medline; Ovid Embase; Ovid Global Health; and Scopus. Grey literature sources were Google Scholar and the WHO websites, complemented by recommendations from stakeholder consultation. Two independent reviewers screened abstracts and full-texts including documents describing research prioritisation activities. Results were analysed using descriptive statistics and narrative synthesis.

**Results:**

We identified 125 publications presenting priority setting activities on 17 high-consequence pathogens published between 1975 and 2022. Most (62%) were related to SARS-CoV-2, 5.6% to Ebola virus and 5% to Zika virus. Three different broad approaches to setting priorities were identified, most (53%) involved external consultations with experts. Few (6%) indicated plans to monitor progress against set priorities.

**Conclusions:**

Our results highlight the diversity in research prioritisation practice in the context of high-consequence pathogens and a limited application of the existing standards in health research prioritisation. An increased uptake of these standards and harmonisation of practice may improve quality and confidence and ultimately improve alignment of funded research with the resulting priorities.

**Supplementary Information:**

The online version contains supplementary material available at 10.1186/s12916-025-03973-8.

## Background

Prioritisation of health research to address key knowledge gaps is essential for the effective allocation of scarce resources. The process of priority setting, however, presents a variety of ethical, methodological and financial challenges [1, 2]. During outbreaks, the complexity associated with research prioritisation is compounded by the need for a rapid response.

Despite the recognised need and benefit of research prioritisation, there are varying standards for developing and reporting research priority agenda, and a lack of consensus on what constitutes best practice in health research priority setting across publications and practice. Some “common themes of good practice” for priority setting in global health have been described, including a need to take cognisance of context; inclusivity of stakeholders involved; advanced planning for implementation of research priorities; and embedding progress monitoring into priority setting processes [2].

Priority setting activities undertaken in advance of an outbreak aim to identify areas for research that can either be undertaken in preparedness (prior to the outbreak’s occurrence) or be rapidly activated when an outbreak occurs. Certain types of research can only be undertaken during an outbreak, such as determining which countermeasures are effective [3]. The need for rapid responses during disease outbreaks in contrast to the element of “anticipatory planning” during outbreak preparedness is likely to impact the processes used for priority setting across these contexts, although both require methodologic robustness.

Methodologies for priority setting for the global [4], regional [5, 6] and national [7] levels have been described. These include systematic processes to generate ordered lists of priorities against specified criteria such as the Child Health and Nutrition Research Initiative (CHNRI) [8] and consensus-based priority setting as described in the Three-Dimensional Combined Approach Matrix (3D CAM), which incorporates values on equity, public health and institutional considerations into the priority setting process [9]. Other reviews have described the approaches currently used for health-research prioritisation [10]. This review aims to map application of these methods, and others, to prioritisation of research associated with high-consequence pathogens.

### Rationale for this review

Despite the availability of broad guidance on health research priority setting, there is limited understanding of the context-specific elements or evidence on the application of these in relation to preparedness and response to disease outbreaks. This scoping review seeks to address this gap and has the following specific objectives:

1. To map published research prioritisation exercises for high-consequence pathogens.
2. To provide a descriptive analysis of approaches used in priority setting for preparedness and response research for high-consequence pathogens.
3. To identify and describe assessment processes for monitoring progress on prioritised research areas.

The need to undertake this scoping review emerged from work undertaken to learn lessons from research prioritisation during the COVID-19 pandemic with the Global Research Collaboration for Infectious Diseases Preparedness (GloPID-R), a global alliance of funders of research on infectious disease threats [11].

## Methods

This scoping review follows the Joanna Briggs Institute’s methodology guidelines for scoping reviews [12] and the Arksey and O’Malley framework [13]. The study protocol is published on Open Research Europe [14].

### Search strategy and selection criteria

A search strategy (Additional file 1: Sect. 1) was developed with support from an information specialist and piloted before being finalised. We searched the following: *Ovid Embase; Ovid Medline; Ovid Global Health; Scopus; the WHO Global Index Medicus*, up to 16 Sept 2022. The search strategies used text words and relevant indexing to retrieve relevant literature about research prioritisation for preparedness and response to outbreaks of (OR) high-consequence pathogens, using additional search terms for outbreaks from the search strategy for the Cochrane review by Pollock et al. (2020) [15]. Grey literature was obtained from searching *Google Scholar* and WHO websites (including the WHO Institutional Repository) on 8/11/2022 and 21/04/2023 respectively. For completeness, we undertook a stakeholder consultation as recommended by Arksey and O’Malley framework [13]. The stakeholders involved were invited from an existing working group (GloPID-R Research in LMICs working group) comprising multidisciplinary representatives of an international group of research funders, who had undertaken work to distil lessons learnt from developing and applying research priorities during the COVID-19 pandemic [16]. The group shared recommended reports and provided references on potential studies to include in the review. After deduplication using *Deduklik*, results from the database search and stakeholder consultation were imported into *Rayyan* for screening. Results from the grey literature search were imported and screened in *Microsoft Excel*.

In this review, high-consequence pathogens are defined as “infectious disease pathogens which cause diseases in humans with the potential to cause outbreaks associated with devastating morbidity and mortality” [14]. We included the 27 pathogens classified as such by either the World Health Organization (WHO) [17], Efficient response to highly dangerous and emerging pathogens at the European Union level (EMERGE) [18], European Centre for Disease Control (ECDC) [19] or Association of Southeast Asian Nations (ASEAN) [20] as detailed in the scoping review protocol [14]. We did not identify any regional priority pathogen lists for the Africa region or the Americas at the time this review was initiated. However, in February 2023 a report ranking epidemic-prone disease threats in Africa was published by the Africa Centres for Diseases Control and Prevention (Africa CDC) [21].

We included published journal articles, policy papers and publicly available reports, commentaries and roadmaps of global, regional and national scope on research prioritisation in preparedness for and in response to outbreaks of high-consequence pathogens. We did not exclude reports based on study design, publication date or language. We excluded records focused solely on antimicrobial resistance (AMR) unless related to any of the included pathogens.

Two reviewers independently screened articles by title and abstract followed by full-texts. The first 100 results of *Google Scholar* and WHO websites searches were screened. Disagreements were resolved through discussion with a third reviewer.

### Data charting

Data charting, validation, processing and analysis were completed in *Microsoft Excel*, using a charting table (Additional file 1: Table 2) which was initially piloted on a sample of 15 studies and revised according to feedback from the coding team. Standardisation of data extracted was facilitated by regular discussions among the coding team during the process and independent validation of the extracted data.

Data items are categorised into five domains: mapping activities for research prioritisation; identifying priority setting approaches; and, detailing processes for monitoring and evaluation (M&E).

### Data analysis

Descriptive analyses involved calculating and comparing the frequencies of categorical variables extracted using *Microsoft Excel*. Qualitative data coded to “communication plan” and “monitoring and evaluation activities” were categorised and described. The duration of priority setting activities and duration of validity of the priority agenda were summarised using descriptive statistical measures using *STATA*.

For the outbreaks which were declared Public Health Emergencies of International Concern (PHEICs), data on the dates of publication of priority setting activities were compared to dates of PHEIC declaration. The time (in days) from PHEIC declaration to publication of priority agenda was determined for each disease (H1N1, Ebola, Zika, COVID-19, mpox) by calculating the difference between the date of publication and the date of PHEIC declaration. Documents without a precise publication date were excluded from this analysis.

### Amendments to the published protocol

The scoping review adhered to the published study protocol, except for one amendment to the sources of grey literature (exclusion of Overton) and the exclusion of backwards citations review of included articles. These amendments were necessitated by resource constraints in the review team due to the high volume of articles identified from the existing sources. To offset potential limitations introduced by this change on the breadth of policy documents assessed, we introduced a search of the WHO websites and input from our expert consultation group (GloPID-R Research in LMICs Working Group).

## Results

**Fig. 1:**
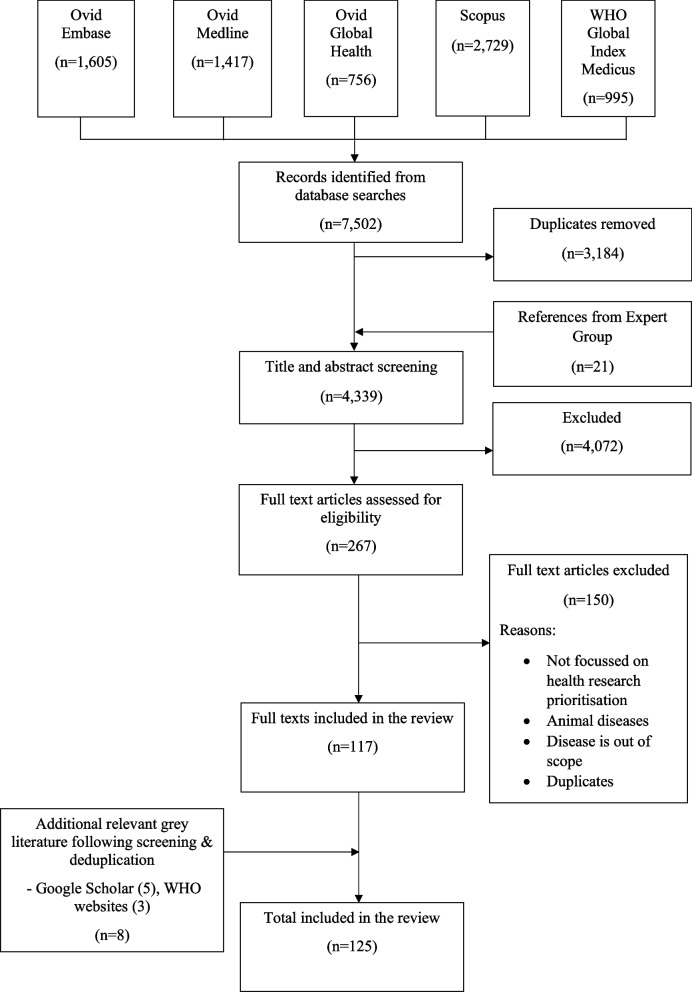
PRISMA flow diagram showing the process of literature search and selection for this study

We identified 125 articles and reports that met the eligibility criteria. Most were journal articles, with only 18 reports. The results are presented following the Preferred Reporting Items for Systematic Reviews extension for Scoping Review in Fig. 1 and included studies are shown in Additional file 1 (Table 3) [3–6, 22–140].

### Scope of research prioritisation publications on high-consequence pathogens

**Fig. 2:**
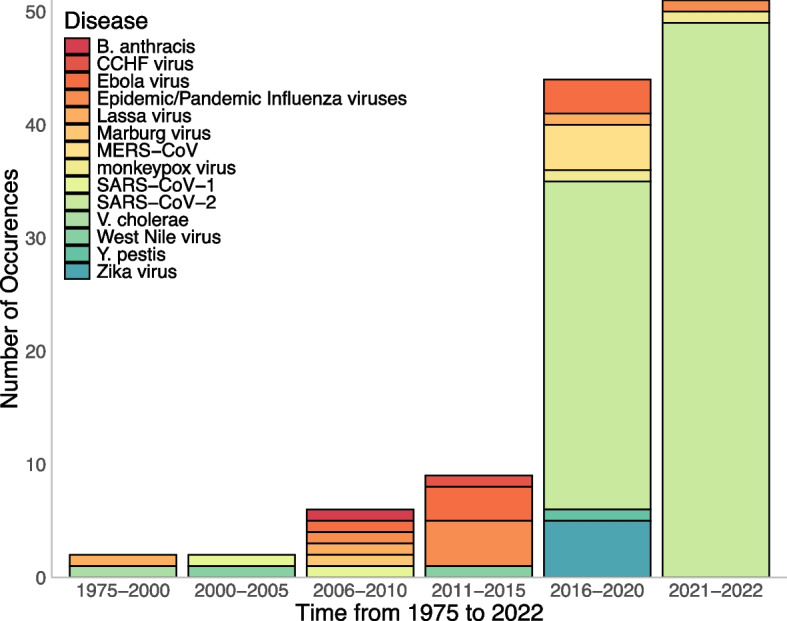
Distribution of research prioritisation publications identified categorised by pathogen focus over time (1975–2022). **Some publications focussed on more than one high-consequence pathogen. Publications focussed on epidemic/pandemic influenza virus and years of publication were as follows: Pandemic Influenza A - (H1N1; 2009, 2011, 2012, 2015, 2021) (H3N2; 2021); Highly Pathogenic Avian Influenza H5N1 (2011, 2015), Low Pathogenic Avian Influenza H7N9 (2013); Swine Influenza A (2011)

We identified research prioritisation activities focussing on 17 of the 27 pathogens within the scope of the review (Fig. 2).

The majority focussed on SARS-CoV-2 (*n* = 78) [3–6, 22–93], followed by Ebola virus (*n* = 7) [94–100], epidemic/pandemic Influenza viruses (*n* = 6) [101–106] and Zika virus (*n* = 5) [107–111]. Publications on the epidemic/ pandemic influenzas focused on Pandemic Influenza A (*n* = 5) [101–103, 105, 106], Highly Pathogenic Avian Influenza A (*n* = 2) [102, 106], Low Pathogenic Avian Influenza (*n* = 1) [104] and Swine Influenza (*n* = 1) [106]. Nine [97, 112–119] were broad in scope and focussed on multiple epidemic/pandemic influenza pathogens. Seven [112–118] of these focused on “Disease X” which recognises “that a serious international epidemic could be caused by a pathogen currently unknown to cause human disease” [17].

**Fig. 3:**
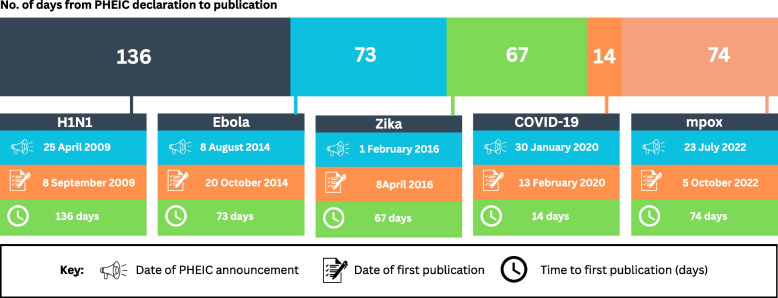
Bar chart comparing dates of the publication of research prioritisation activities and PHEIC declaration. **No identified publications focussing on Ebola 2018 outbreak

All records included a year of publication and 90% (112/125) [3–6, 22–33, 35–40, 42–55, 57–84, 86, 87, 91, 105, 107–135] of them included an exact publication date (day/month/year). The earliest record identified was published in 1975 (Fig. 2). The distribution of publications shows a clear skew post-2020 as 89% (*n* = 78/88) [3–6, 22–93] of post-2020 publications focussed on SARS-CoV-2. The results from comparing the date of the published document to the date of a PHEIC declaration for the relevant outbreaks (*n* = 97/125) [3–6, 22–96, 98–103, 105–111, 125, 131, 134] are shown in Fig. 3. In order of the shortest time to publication of a priority setting activity from the PHEIC declaration, publications focussed on COVID-19, Zika, Ebola, mpox and H1N1 respectively.

### Approaches for developing research priorities

#### Approaches to identify research priorities

**Fig. 4:**
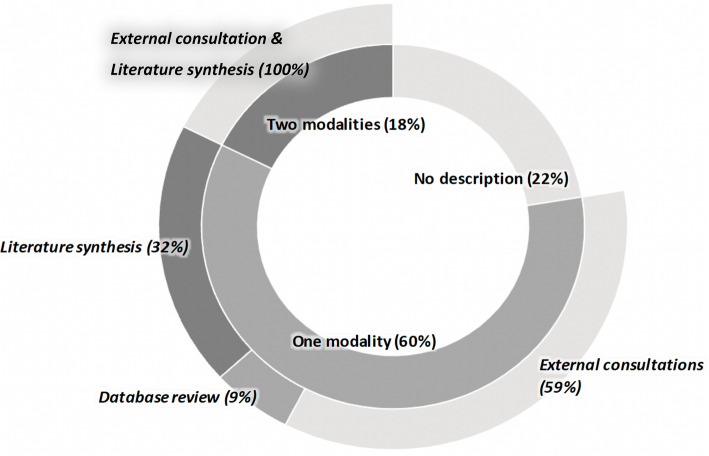
Sunburst visualisation of the approaches used in the identification of research priorities for high-consequence pathogens

Figure 4 shows the distribution of the prespecified approaches used to identify research priorities in the publications analysed. Of the 125 publications, 78% (*n* = 97/125) [3–6, 22–39, 42–47, 49, 51, 56, 58–60, 62–64, 66–70, 73–75, 77, 80, 82, 84–91, 93–95, 97, 98, 101, 102, 104, 107, 109–118, 120–122, 124–129, 131, 132, 135, 136] described the approaches used to identify priorities. Of the methods used, we identified three key modalities: external consultations, literature synthesis and database reviews (assessments of research gaps from various databases which collate information on research). Either one modality (*n* = 75) or two modalities (*n* = 22) were used. The priority setting activities that used one modality involved external consultations (*n* = 44) [3, 5, 6, 22, 33, 46, 47, 49, 53, 56, 59, 66, 67, 69, 70, 73, 75, 80, 82, 84, 86, 91, 93, 94, 97, 98, 101, 102, 105, 106, 111, 112, 114, 116, 124, 125, 127, 131], database review (*n* = 7) [24–28] or literature synthesis (*n* = 24) [23, 30, 31, 35, 37, 43, 44, 54, 55, 60, 62, 63, 68, 77, 81, 95, 104, 107, 109, 113, 128, 129, 135]. Those using two modalities involved external consultations and literature synthesis (*n* = 22) [4, 29, 32, 34, 38, 39, 42, 45, 51, 52, 58, 64, 85, 110, 117, 118, 120, 122, 126, 132, 136].

The priority setting activities involving external consultations (*n* = 66) were via one or more of the following: interviews (*n* = 9) [39, 58, 85, 87, 88, 98, 116, 121, 122], surveys (*n* = 25) [3, 5, 6, 22, 34, 39, 42, 49, 56, 58, 64, 67, 69, 70, 73, 75, 80, 82, 84, 87, 88, 110, 112, 116, 121] or meetings (*n* = 50) [3–6, 22, 29, 33, 38, 42, 45, 47, 49, 51, 52, 56, 59, 64, 66, 69, 70, 80, 84–87, 89–91, 94, 97, 98, 101, 102, 105, 106, 110–112, 114, 118, 120–122, 124–127, 131, 132, 136] and took place online (*n* = 26) [3, 5, 6, 32, 33, 38, 39, 46, 49, 59, 64, 69, 70, 73, 75, 80, 84–87, 93, 105, 112, 114, 120, 122], in-person (*n* = 11) [4, 56, 98, 106, 110, 124–127, 132, 136] or both (*n* = 6) [22, 89, 97, 101, 118, 121]. The mode of consultation was not stated in 23 publications [29, 34, 39, 42, 45, 47, 51–53, 58, 66, 67, 82, 88, 90, 91, 94, 102, 111, 115–117, 131] using external consultations. Where literature synthesis was done (*n* = 46), most used literature reviews (*n* = 32) [4, 31, 34–36, 38, 39, 42–45, 51, 52, 54, 55, 58, 60, 63, 64, 81, 85, 95, 104, 107, 109, 121, 122, 128, 129, 132, 135, 136] followed by systematic literature reviews (*n* = 10) [23, 29, 30, 37, 62, 77, 110, 113, 120, 126] and scoping reviews (*n* = 4) [32, 68, 117, 118].

In 77% (*n* = 96/125) [3–6, 22, 24–28, 30, 32–34, 36, 38–40, 42, 43, 45–53, 55–59, 61, 64, 69–91, 93, 95–99, 101, 102, 105, 106, 110–118, 120–128, 130–132, 134, 136–138] of publications, priority setting involved the engagement of stakeholders. Among these, the broad term “expert” occurred most frequently (*n* = 85/96) [3, 4, 6, 22, 24–28, 30, 32–34, 36, 38–40, 42, 43, 45–53, 55–59, 61, 64, 69–72, 76–81, 83–85, 87, 89, 93, 95–97, 99, 101, 102, 105, 106, 110–116, 118, 120–128, 130–132, 134, 136–138] followed by “researchers” and “policy makers”.

#### Named specific approaches to identify research priorities

For seven publications specific prioritisation methodologies were named as being applied alone or in combination in the priority setting processes. These include Child Health and Nutrition Research Initiative (*n* = 5) [5, 49, 70, 116, 121], Essential National Health Research (*n* = 2) [5, 98], Rapid Research Needs Appraisal (*n* = 1) [120] and Combined Approach Matrix (*n* = 1) [98]. Other methods mentioned are Delphi method (*n* = 8) [46, 53, 74, 93, 110, 115, 117, 118] and Nominal Group technique (*n* = 1) [122].

#### Approaches to grouping research priorities

Priority setting activities involved multiple stages in most publications. For 38% (*n* = 48/125) [33, 36, 40, 41, 43, 44, 47, 48, 50, 52, 54, 57, 61, 65, 66, 71, 76–78, 81–83, 89–92, 94, 99–101, 103–106, 108, 109, 119, 123, 125, 128, 129, 133–136, 138–140], there was no description of the method for grouping the priorities identified. Twenty-six percent (*n* = 32/125) [5, 24–28, 30, 32, 34, 45, 46, 49, 53, 60, 70, 72, 73, 79, 84–86, 95–97, 111, 116, 121, 127, 130, 131] of publications grouped priorities based on pre-determined categories, 23% (*n* = 29/125) [23, 29, 31, 35, 39, 51, 55, 56, 59, 62–64, 67–69, 74, 75, 80, 93, 98, 107, 113–115, 117, 118, 124, 132, 137] were based on emergent areas and 13% (*n* = 16/125) [3, 4, 6, 22, 37, 38, 42, 58, 87, 88, 102, 110, 112, 120, 122, 126] used a blend of the two approaches.

#### Approaches for shortlisting research priorities

For 76% (*n* = 95/125) [4, 23–33, 35–37, 39–44, 47, 48, 50–52, 54–57, 59–63, 65–68, 71, 72, 76, 77, 79, 81–83, 85–87, 89–92, 94–97, 99–109, 111, 113, 114, 118–120, 123–140] of publications, there was no description of approaches used in shortlisting priority areas. A variety of approaches to shortlisting priorities were identified including ranking (*n* = 27) [3, 5, 6, 22, 34, 38, 46, 49, 53, 58, 64, 69, 70, 73–75, 78, 80, 84, 88, 98, 110, 112, 116, 117, 121, 122], selection by experts (*n* = 2) [45, 115] and content analysis to draw out themes (*n* = 1) [93]. The ranking of priority lists was based on scoring, voting or rating (*n* = 21) [3, 5, 6, 8, 22, 34, 38, 46, 49, 58, 69, 70, 73–75, 80, 84, 88, 110, 116, 117], expert judgement (*n* = 2) [98, 122] and frequency of occurrence (*n* = 3) [53, 64, 112] and digital software outputs (*n* = 1) [78]. In 33% (*n* = 9/27) [6, 49, 58, 80, 84, 112, 116, 117, 121] of the projects applying ranking, weighting was done to further refine priorities identified to give an indication of areas of higher or lower priority.

Multiple criteria were applied in the identification of research priorities for high-consequence pathogens in this review. These were applied both where priorities were shortlisted from a number of options (and formed the basis of the shortlisting process) or were mentioned as the criteria for priority setting (where no shortlisting processes were described). The most commonly mentioned criterion was “need” which was used in 70% (*n* = 87/125) [3–6, 22–31, 34–39, 41–43, 45–54, 56–59, 62, 64, 66, 69–71, 73, 75, 76, 79, 83–88, 91, 94, 96–98, 100–102, 105, 107–113, 115, 116, 118–122, 124, 126, 127, 131, 135, 136, 138–140] of publications. Others were as follows: “research gaps”, “feasibility”, “impact”, “efficiency” and “effectiveness”, “answerability” and “relevance”. In 14% (*n* = 17/125) [61, 67, 68, 72, 78, 80, 82, 90, 92, 103, 104, 113, 123, 129, 130, 133, 134], no criteria for selection of priorities was described.

#### Duration of research prioritisation activities

The duration for priority setting activities was stated in 24% (*n* = 30/125) [3–6, 22, 24, 28, 45, 53, 56, 58, 60, 70, 73, 74, 84, 87, 101, 110, 116, 120, 121, 134] of publications. These focussed on outbreak response (*n* = 17) [4, 24–28, 45, 53, 56, 58, 70, 73, 85, 86, 134], preparedness (*n* = 5) [87, 101, 110, 120, 121] or both (*n* = 8) [3, 5, 6, 22, 60, 74, 84, 116]. On average, priority setting took 33 months (SD ± 24 months). When publications solely focussing on preparedness (mean = 448 months) or response (mean = 327 months) respectively were compared, there was no statistically significant difference between the mean duration for undertaking priority setting activities (*t*(20) = − 0·78, M_diff = 1·21, *p* = 0·45, 95%CI [− 2·03, 4·44]).

Description of approaches: External consultations—engagement of individuals/groups/organisations in the priority setting process; Database reviews—assessments of research gaps from various databases which collated information on research; Literature synthesis—reviews of published studies.

#### Research priorities for high-consequence pathogens

**Fig. 5:**
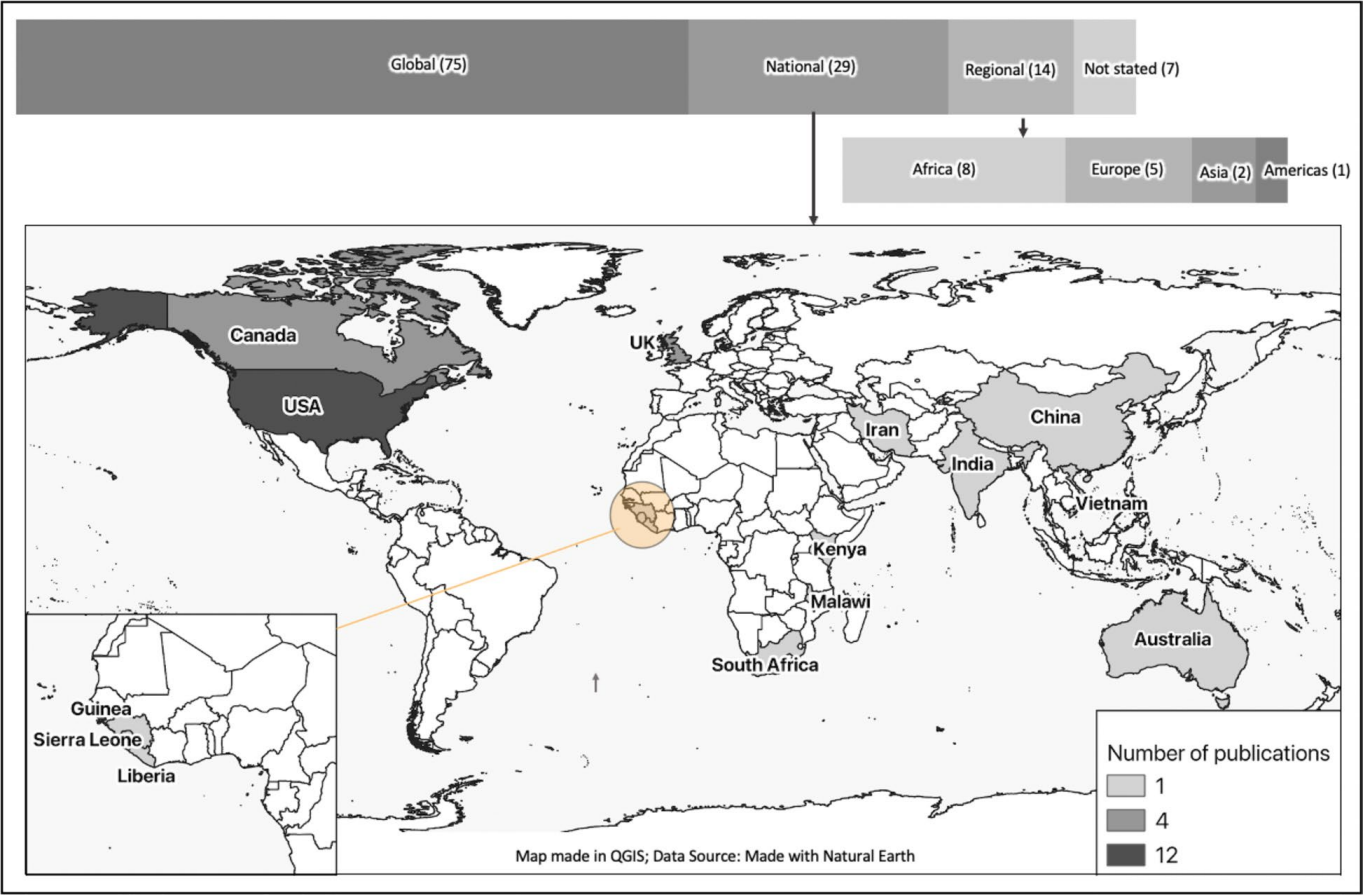
Geographical areas targeted in publications on research prioritisation for high-consequence pathogens

The intent for the priority setting was identified as “preparedness” (*n* = 27) [64, 83, 87, 93, 96, 99, 101–103, 107, 108, 110, 114, 117, 118, 120, 121, 123, 125, 126, 132, 136, 138, 140], “response” (*n* = 55) [4, 23–28, 30, 31, 33, 34, 39, 41, 44, 45, 48, 49, 51, 53, 54, 56, 58, 61–63, 65, 69, 71, 73, 75, 77, 79–82, 85, 86, 88, 90, 91, 94, 100, 104, 105, 109, 111, 128, 131, 133, 134] or both (*n* = 43) [3, 5, 6, 22, 29, 32, 35, 38, 40, 42, 43, 46, 47, 50, 52, 55, 59, 60, 66, 68, 72, 74, 78, 84, 89, 92, 95, 106, 112, 113, 115, 116, 119, 122, 124, 127, 129, 130, 135, 137]. Figure 5 depicts the geographical distribution of areas targeted by the priority setting exercises identified and shows most (*n* = 75) [3, 4, 24, 34, 36, 37, 40, 43, 46, 47, 50, 52, 55, 61–64, 68, 71, 72, 74, 77, 79–83, 85–87, 89, 91, 93, 95, 97, 101, 106, 108, 111–113, 117, 118, 120, 121, 123, 125, 128, 130, 131, 133, 135, 137, 138, 140] research priorities set were of global scope. Others focussed on the regional (*n* = 14) [5, 6, 41, 42, 50, 51, 84, 94, 99, 102, 109, 110, 115, 132] or national level (*n* = 29) [22, 38, 39, 45, 48, 49, 56, 60, 65, 67, 69, 73, 88, 90, 92, 98, 103, 105, 114, 116, 122, 124, 129, 131, 136, 139]. In seven publications [23, 35, 44, 78, 100, 104, 119], no geographical target was identified. Africa was identified as the primary focus in regional research priority setting agenda, followed by Europe, Asia and the Americas. The majority of national level priority setting activities were for the USA, Canada or the UK.

In 82% (*n* = 102/125) [3–6, 22–30, 33, 35–37, 40–42, 44, 46–48, 50, 51, 54–56, 58–72, 74, 76, 78, 79, 81, 83–87, 89–102, 104–112, 114–116, 119–135, 137–139] of publications, specific target populations for the set priorities were not stated. Women, children, pregnant women and adolescents were most commonly listed in the 19% presenting target populations.

Regions targeted in the 125 publications identified. Some publications focussed on more than one level of priority setting or location. Two publications focussed on low- and middle-income countries (LMICs).

#### Monitoring and dissemination of priorities set

Only eight (6%) [24–28, 101] of the publications included plans to review progress against the priorities. Seven [24–28] were in a series of living reviews for COVID-19 research which were revised quarterly. Only three publications [87, 101, 121] stated a duration of validity of the priorities set. Ninety percent (*n* = 113/125) [3–6, 22, 23, 29–31, 33–41, 43–89, 91–100, 102–117, 119–140] of publications did not list a plan for dissemination of the identified research priorities. For the publications which did, dissemination was via websites (*n* = 7) [24–28], stakeholder engagement (*n* = 3) [32, 42, 90], information briefs (*n* = 1) [118] or unspecified (*n* = 1) [101].

## Discussion

Our results show the breadth of research prioritisation activities focussing on high-consequence pathogens over a 47-year period (1975–2022). Priority setting activities increased steadily over time and rose exponentially from 2020 to 2022, attributable to the COVID-19 pandemic. Given that SARS-CoV-2 was a novel pathogen and the large scale of the COVID-19 pandemic, it is unsurprising that many priority setting activities focussed on this disease. Similarly, in the years preceding 2020, pathogens responsible for the largest epidemics dominated the research prioritisation agenda.

The trends show an increase in prioritisation activities following the major outbreaks caused by H1N1 virus (2009), Ebola Virus (2014), Zika Virus (2016) and SARS-CoV (2019). While H1N1 and SARS-CoV-2 were novel pathogens, Zika and Ebola viruses, were known high-priority pathogens though priority setting activities for these viruses largely did not occur until they caused major outbreaks. This finding supports the view that reactiveness rather than proactiveness drives action in this field.

The lack of proactive considerations is further supported by the lack of research prioritisation publications identified on ten of the pathogens within the scope of this review, each categorised as “high priority” regionally and/ or globally, though for which no research prioritisation agenda were identified. Some priority setting publications identified did, however, cover “Disease X” [17] and could indicate an awareness of the epidemic/pandemic threats posed by newly emerging pathogens.

Outbreaks of six of the high-consequence pathogens in scope for this review (H1N1 virus, Ebola virus, Zika virus, SARS-CoV-2 and monkeypox virus) were declared PHEICs. Our analysis showed a decreasing trend in time from the declaration of these outbreaks as PHEICs to the publication of the research prioritisation agenda from the 2009 H1N1 outbreak to the 2019 COVID-19 outbreak. Mpox was the exception among the data reviewed which showed an increase in time to publication compared with the preceding COVID-19 pandemic. No publications related to the 2018 Ebola outbreak, which was also declared a PHEIC, were identified in this review.

These results could indicate faster initiation of research responses for successive PHEICs, with the exception of mpox. However, it is challenging to compare outbreak responses given that each of these outbreaks occurred under unique global/regional conditions with responses likely to have been influenced by multiple factors including biological, political and social factors that are beyond the research responses [141].

### Approaches for developing research priorities

We identified a variety of approaches used for priority setting for research on high-consequence pathogens. Our findings are consistent with the diversity in practice discussed in the literature [142]. Where details were available, we found that priority setting often involved three stages: the identification of priority areas, grouping these areas and shortlisting the priority areas.

Identifying priority areas typically took three forms: external consultations, literature synthesis and database reviews. External consultations via meetings, interviews and surveys were the most frequently used approach to identifying research priorities for high-consequence pathogens. Although all three approaches promote the extraction of a range of views from various stakeholders, meetings have the additional benefit of promoting collaborative decision making through the interaction of stakeholders. The rapid advancements in online communication over the past decades have likely contributed to enhanced stakeholder interactions and the inclusion of stakeholders which hitherto had limited opportunities to participate in external consultations. This was witnessed during the COVID-19 pandemic where there are examples of increased participation in online consultations despite the restrictions to movement imposed in response to the pandemic [143, 144]. While literature synthesis identifies gaps in the published evidence, external consultations distil gaps from stakeholder knowledge and experience. Database reviews focussed on assessing research funding allocations for identifying research gaps in priority areas at the time of funding commitments.

The majority of publications did not report a process for further shortlisting the identified priorities. Therefore, there is a risk that the priority setting activities could lead to the creation of a “menu” of options with limited ability to inform decision making given their lack of specificity on the actual weighting to be given to the priorities identified.

Only seven publications applied any of the well-known health research priority setting methodologies suggesting that these approaches might not be widely applied in this context. Our findings do not show any distinction between approaches used in preparedness priority setting or priority setting in response mode. However, we identified the Rapid Research Needs method which is specifically designed to rapidly identify evidence gaps using expedited review processes which effectively utilise work patterns across global time zones [120]. Another notable method identified for anticipatory planning for outbreaks was a scenario-based method to model future event outcomes for predicting research needs [87].

The inclusion of diverse perspectives is recommended among the good practices for health research priority setting [2, 142]. While various experts were involved in the priority setting exercises identified, the majority did not specify the meaning of “experts” or a rationale for their involvement in these processes. A limited understanding of which perspectives were sought in the development of research priorities could call into question the validity of priority setting outputs. Without representation of the right voices, priority setting activities risk producing agenda which are not truly representative of the research needs of the populations and settings they target. Extensive consultations are likely to be challenging particularly in the context of outbreak response. One way to address this is to pre-position expert groups in advance of disease outbreaks such that they can rapidly be engaged for effective priority setting in the event of an outbreak [16].

Our analysis shows that the average priority setting activities took three months to complete. However, data was available for only 25% of the publications we reviewed and was skewed to those publications focussing solely on outbreak response. This limited data resulted in an inability to make a comprehensive assessment of the durations of priority setting activities for high-consequence pathogens from the publications we reviewed.

The available details on approaches applied to research prioritisation varied across the publications we reviewed and the level of detail provided declined over the stages of priority setting identified. Taken together, the findings from reviewing research priority setting approaches indicate significant variability in the quality of the reporting on approaches used in the identification of research priorities for high-consequence pathogens. Similar findings have emerged from other reviews of health research priority setting in different contexts where the reports of priority setting approaches were found to be sub-optimal [145, 146]. This limited transparency in priority setting may threaten the perceived validity and credibility of priority setting activities and negatively impact uptake and implementation of priority agenda set.

In their 2019 paper, Tong et al. outline a standard framework for reporting on research priorities, the “Reporting guideline for priority setting of health research (REPRISE)” [147]. REPRISE was developed to address the lack of a standard reporting guide for health research prioritisation. Uptake of these guidelines could strengthen the effectiveness and impact of prioritisation activities in health research.

### Research priorities for high-consequence pathogens

The purpose of priority setting identified in the publications reviewed was preparedness, response or both. The finding of most publications being set in response mode supports the previous findings of this review which suggests responses to disease outbreaks appear to be more reactive than proactive. Notably, some publications were set for both preparedness and response suggesting that even during acute epidemic responses, the priorities set were forward-looking, factoring in preparedness for future epidemics and pandemics (concerning the ongoing outbreak or outbreaks of other high-consequence pathogens).

The results also show prioritisation activities for high-consequence pathogens targeted multiple levels and were of global, regional or national scope. The majority of publications focussed on the global level. While this is to be expected among studies focusing on COVID-19, the dominant focus of publications where COVID-19 was not the focus, remained at the global level. A possible explanation could be that the pathogens included in this review were based on global/regional assessments of pathogens posing global health threats. Further, it is possible that global level prioritisation activities would be more likely to be published and hence more accessible through literature searches than prioritisation targeting the other levels. Given the varying contexts and needs across levels, research priorities set, regardless of the level targeted, need to be adapted to the settings and populations in which they will ultimately be implemented. In particular, the needs of vulnerable populations and populations often in the minority should be considered. An assessment of target populations for the priorities set is limited by insufficient data since only 19% of the publications we reviewed stated specific populations targeted by the research agenda.

### Monitoring and dissemination of priorities set

Beyond the publication of the outputs of priority setting as the reports and journal publications reviewed, we found limited information on dissemination plans for the research priorities set. A lack of awareness of prioritisation activities undertaken could lead to duplicative prioritisation efforts and negatively impact implementation of the priorities set.

The examples of dissemination activities identified include publication on websites, direct engagement with stakeholders and publication of information briefs. A specific search focussing on dissemination activities might have yielded further examples as those listed here were derived from reviewing dissemination activities noted in those publications on high-consequence pathogens we identified. The modes of communication selected are likely to be influenced by which stakeholders are targeted for implementing the priority setting agenda. To facilitate action on the outputs of research priority setting activities, it is important that the appropriate modes of communication which are suitable for the audiences involved are applied.

Monitoring progress against priorities set is important for assessing the impact of the research priorities and the identification of persistent research gaps and new areas for prioritisation. This was exemplified in the COVID-19 pandemic where tracking funded research projects and their alignment to global and regional research priorities was crucial in informing research funding decisions [144].

We found limited information on plans to monitor progress against priorities set for high-consequence pathogens. Embedding M&E processes into priority setting activities is among the recommended practices outlined in the literature. However, there is limited guidance on how individuals/groups who set research priorities can practice this. Available resources, perspectives considered for M&E processes, timing, the role of individuals setting priorities in their implementation and availability of information on the research being undertaken are likely to be among the important factors to consider. There are also likely to be factors (which go beyond the availability of research priorities) that influence research funding decisions. This poses a challenge to accessing the impact of the results of research prioritisation activities. The new Pandemic PACT programme aims to partially address this [148].

### Limitations

Several limitations need to be considered in the interpretation of findings from this scoping review. Firstly, the high-consequence pathogens included in this study were obtained from global and regional lists of priority pathogens, where available. Therefore, priority lists from the WHO, EMERGE, ECDC and ASEAN were included. We did not identify any regional priority pathogen lists for the Americas or the Africa region at the time this review was initiated. Further, pathogens prioritised at the national or local level were not considered.

Some analyses in this review utilised dates of publication of research prioritisation activities for making inferences about the timeliness of their implementation during outbreak responses. We acknowledge this approach does not consider other broader activities (apart from journal publications and reports) which might have been undertaken to disseminate information on the priorities set.

## Conclusions

Our scoping review identified publications outlining research priorities relating to 17 high-consequence pathogens, the majority of which were set in response to major outbreaks. There was a lack of consistency in the approaches and methods used for prioritisation, level of detail reported in priority setting publications and limited information on plans to monitor progress made against the priorities set.

Our results show that priority setting for preparing and responding to outbreaks of high-consequence pathogens is a complex endeavour. Variations in the approaches taken to set priorities are likely to be dependent on the various contexts and rationales for which priorities were set. Greater application of existing standards for research priority setting may improve the confidence and trust in the priority setting process and their likelihood of implementation.

## Supplementary Information

Additional file 1. Search strategy, Data extraction table, Charateristics of includes studies & References of included files.

## Data Availability

No datasets were generated or analysed during the current study.

## Abbreviations

3D CAM: Three-Dimensional Combined Approach Matrix
AMR: Antimicrobial resistance
ASEAN: Association of Southeast Asian Nations
CHNRI: Child Health and Nutrition Research Initiative
EMERGE: Efficient response to highly dangerous and emerging pathogens at EU level
Europe CDC: European Centre for Disease Prevention and Control
GloPID-R: Global Research Collaboration for Infectious Diseases Preparedness
LMICs: Low- and middle-income countries
Pandemic PACT: Pandemic Preparedness Analytical Capacity and funding Tracking
PHEIC: Public Health Emergency of International Concern
REPRISE: Reporting guideline for priority setting of health research
WHO: World Health Organization
