# Supplementary material for: Research prioritisation in preparedness for and response to outbreaks of high-consequence pathogens: a scoping review
Source: BMC Med. 2025 Mar 10;23:147. doi: 10.1186/s12916-025-03973-8 (PMC11892158; doi:10.1186/s12916-025-03973-8)
Supplement: Supplementary file 1 — Additional file 1. Search strategy, Data extraction table, Charateristics of includes studies & References of included files. [file 12916_2025_3973_MOESM1_ESM.pdf]

Table of Contents

1. Search Strategy for Databases included in Scoping Review..1
2. Data Extraction Table for Scoping Review..7
3. Characteristics of the included publications 9
4. References of included studies..20

1. Search Strategy for Databases included in Scoping Review

Database: Embase 1974 to present
Search Strategy:
----
1 Ebola hemorrhagic fever/ or Marburg hemorrhagic fever/ or Marburg virus/ or Marburgvirus/ (8657)
2 lassa fever/ or lassa virus/ (1983)
3 Crimean Congo hemorrhagic fever/ or Crimean-Congo hemorrhagic fever virus/ (1821)
4 Rift Valley fever/ or rift valley fever virus/ (1584)
5 (ebola* or ebov or marburg* or lassa* or CCHF or CCHVF or "crimean-congo*" or "congo virus" or (crimean adj2 (hemorrhagic or haemorrhagic)) or "rift valley*" or RVF or RVFV).ti,ab,kw. (22432)
6 exp coronavirinae/ or exp Coronavirus infection/ or severe acute respiratory syndrome/ (303109)
7 ("middle east* respiratory syndr*" or MERS-CoV or "novel CoV*" or "novel betacoronavirus" or coronavirus* or covid* or ("middle east" adj3 (cov or betacoronavirus*)) or (MERS adj3 (cov or betacoronavirus*)) or "mers-coronavirus" or "mers cov" or merscov or "wuhan flu" or 2019-nCoV).tw. (308284)
8 exp West Nile virus/ (3385)
9 ("west nile" or "egypt 101").ti,ab,kw. (10079)
10 exp "influenza a virus (h1n1)"/ or "influenza a virus (h5n1)"/ or "influenza a virus (h3n2)"/ or "Influenza A virus (H7N9)"/ (9140)
11 (H1N1 or H5N1 or H3N2 or H7N9).ti,ab,kw. (35847)
12 exp henipavirus/ or exp Henipavirus infection/ (1864)
13 (nipah or hendra).tw. (1648)
14 African swine fever virus/ or African swine fever/ or ("african swine" or "ASF virus" or ASFV or "wart hog disease virus*").ti,ab,kw. (2856)
15 monkeypox/ or monkeypox virus/ (1520)
16 (monkeypox or "monkey pox").tw. (1544)
17 exp Zika virus/ or Zika fever/ (12369)
18 (zika* or zikv).ti,ab,kw. (12744)
19 ("disease x*" or "pathogen x*").ti,ab,kw. (50757)
20 exp poliomyelitis/ or polio*.ti,ab,kw. (27786)
21 smallpox/ or Smallpox virus/ (6859)
22 (smallpox* or "small pox*" or variola).ti,ab,kw. (6700)
23 exp brucellosis/ (11949)
24 (brucellosis or brucelloses or brucella).ti,ab,kw. (16567)
25 Q fever/ (5045)
26 ("q fever*" or "Coxiella burnetii" or coxiellosis or "query fever*").ti,ab,kw. (6008)
27 melioidosis/ (3205)

28 (melioidosis or pseudomallei or "whitmore* disease*" or "nightcliff gardener* disease*" or pseudoglanders or "paddy-field disease" or "paddyfield disease").ti,ab,kw. (4330)
29 tularemia/ (3429)
30 (Tularaemia* or tularemia* or "Francisella tularensis" or "ohara disease" or "rabbit fever").ti,ab,kw. (4440)
31 cholera/ (10547)
32 cholera*.ti,ab,kw. (31988)
33 exp plague/ (6571)
34 (plague or "black death" or "Yersinia pestis" or "Y. pestis").ti,ab,kw. (10973)
35 glanders/ (213)
36 (Glanders or "Burkholderia mallei").ti,ab,kw. (609)
37 exp anthrax/ (6574)
38 (Anthrax or "Bacillus anthracis" or "B. anthracis").ti,ab,kw. (9521)
39 ("priority pathogen*" or "potential pathogen*" or "re-emerging pathogen*" or "reemerging pathogen*" or "emerging pathogen*").ti,ab,kw. (14648)
40 epidemic/ (123502)
41 pandemic/ (139546)
42 (((health$ or disease$) adj5 (disaster$ or catastrophe$ or crises or crisis)) or epidemic* or pandemic* or outbreak* or out-break* or "public health emergenc*" or "global health emergenc*").ti,ab,kw. (426552)
43 1 or 2 or 3 or 4 or 5 or 6 or 7 or 8 or 9 or 10 or 11 or 12 or 13 or 14 or 15 or 16 or 17 or 18 or 19 or 20 or 21 or 22 or 23 or 24 or 25 or 26 or 27 or 28 or 29 or 30 or 31 or 32 or 33 or 34 or 35 or 36 or 37 or 38 or 39 or 40 or 41 or 42 (868949)
44 research priority/ (4151)
45 ((setting* adj2 priorit*) or (research* adj2 priorit*) or "research strateg*" or "prioriti?ation methodolog*" or (generat* adj2 priorit*) or (develop* adj2 priorit*) or "priority guideline*" or "agenda setting*" or (research* adj1 gap*) or (identif* adj2 priorit*) or "research question*").ti,ab,kw. (61321)
46 44 or 45 (62685)
47 (preparedness or readiness or "in advance of" or "planning for").ti,ab,kw. (385419)
48 infection control/ (96474)
49 prevention/ (288469)
50 (response or control or prevention or addressing or tackle).ti,ab,kw. (7109751)
51 47 or 48 or 49 or 50 (7582501)
52 43 and 46 and 51 (1605)

Database: Medline (Ovid MEDLINE® Epub Ahead of Print, In-Process & Other Non-Indexed Citations, Ovid MEDLINE® Daily and Ovid MEDLINE®) 1946 to present
Search Strategy:
----
1 hemorrhagic fever, ebola/ or lassa fever/ or marburg virus disease/ or rift valley fever/ (8692)
2 exp Filoviridae/ (4399)
3 Lassa virus/ (791)
4 Hemorrhagic Fever Virus, Crimean-Congo/ or Hemorrhagic Fever, Crimean/ (1427)
5 (ebola* or ebov or marburg* or lassa* or CCHF or CCHVF or "crimean-congo*" or "congo virus" or (crimean adj2 (hemorrhagic or haemorrhagic)) or "rift valley*" or RVF or RVFV).ti,ab,kw. (18909)
6 COVID-19/ (186659)
7 middle east respiratory syndrome coronavirus/ or sars virus/ or sars-cov-2/ (141224)
8 Severe Acute Respiratory Syndrome/ or Coronavirus Infections/ (50163)
9 ("middle east* respiratory syndr*" or MERS-CoV or "novel CoV*" or "novel betacoronavirus" or coronavirus* or covid* or ("middle east" adj3 (cov or betacoronavirus*)) or (MERS adj3 (cov or

betacoronavirus*)) or "mers-coronavirus" or "mers cov" or merscov or "wuhan flu" or 2019-nCoV).ti,ab,kw. (288855)
10 West Nile virus/ or West Nile Fever/ (6060)
11 ("west nile" or "egypt 101").ti,ab,kw. (8555)
12 influenza a virus, h1n1 subtype/ or influenza a virus, h3n2 subtype/ or influenza a virus, h5n1 subtype/ or influenza a virus, h7n9 subtype/ (25999)
13 (H1N1 or H5N1 or H3N2 or H7N9).ti,ab,kw. (29472)
14 exp Henipavirus/ or Henipavirus Infections/ (923)
15 (nipah or hendra).ti,ab,kw. (1447)
16 African Swine Fever/ (1334)
17 ("african swine" or "ASF virus" or ASFV or "wart hog disease virus*").ti,ab,kw. (2500)
18 Monkeypox virus/ or Monkeypox/ (939)
19 (monkeypox or "monkey pox").ti,ab,kw. (1571)
20 Zika Virus Infection/ or Zika Virus/ (7329)
21 (zika* or zikv).ti,ab,kw. (10600)
22 ("disease x*" or "pathogen x*").ti,ab,kw. (1992)
23 Smallpox/ (6050)
24 (smallpox* or "small pox*" or variola).ti,ab,kw. (9423)
25 exp Poliomyelitis/ (20474)
26 polio*.ti,ab,kw. (32735)
27 exp Brucellosis/ (13356)
28 (brucellosis or brucelloses or brucella).ti,ab,kw. (18524)
29 Q Fever/ (5157)
30 ("q fever*" or "Coxiella burnetii" or coxiellosis or "query fever*").ti,ab,kw. (6679)
31 exp Burkholderia Infections/ (3899)
32 (melioidosis or pseudomallei or "whitmore* disease*" or "nightcliff gardener* disease*" or pseudoglanders or "paddy-field disease" or "paddyfield disease").ti,ab,kw. (4072)
33 (Glanders or "Burkholderia mallei").ti,ab,kw. (672)
34 Tularemia/ (3542)
35 (Tularaemia* or tularemia* or "Francisella tularensis" or "ohara disease" or "rabbit fever").ti,ab,kw. (4995)
36 Cholera/ (9155)
37 cholera*.ti,ab,kw. (32693)
38 exp Yersinia Infections/ (9655)
39 (plague or "black death" or "Yersinia pestis" or "Y. pestis").ti,ab,kw. (11941)
40 Anthrax/ (4473)
41 (Anthrax or "Bacillus anthracis" or "B. anthracis").ti,ab,kw. (9618)
42 ("priority pathogen*" or "potential pathogen*" or "re-emerging pathogen*" or "reemerging pathogen*" or "emerging pathogen*").ti,ab,kw. (12111)
43 disease outbreaks/ or epidemics/ or pandemics/ (194530)
44 (((health$ or disease$) adj5 (disaster$ or catastrophe$ or crises or crisis)) or epidemic* or pandemic* or outbreak* or out-break* or "public health emergenc*" or "global health emergenc*").ti,ab,kw. (392003)
45 1 or 2 or 3 or 4 or 5 or 6 or 7 or 8 or 9 or 10 or 11 or 12 or 13 or 14 or 15 or 16 or 17 or 18 or 19 or 20 or 21 or 22 or 23 or 24 or 25 or 26 or 27 or 28 or 29 or 30 or 31 or 32 or 33 or 34 or 35 or 36 or 37 or 38 or 39 or 40 or 41 or 42 or 43 or 44 (748505)
46 ((setting* adj2 priorit*) or (research* adj2 priorit*) or "research strateg*" or "prioriti?ation methodolog*" or (generat* adj2 priorit*) or (develop* adj2 priorit*) or "priority guideline*" or "agenda setting*" or (research* adj1 gap*) or (identif* adj2 priorit*) or "research question*").ti,ab,kw. (49347)
47 (preparedness or readiness or "in advance of" or "planning for").ti,ab,kw. (288985)

48 exp Infection Control/ (69544)
49 exp Primary Prevention/ (174936)
50 (response or control or prevention or addressing or tackle).ti,ab,kw. (5503572)
51 47 or 48 or 49 or 50 (5885420)
52 45 and 46 and 51 (1417)

Database: Global Health <1973 to 2022 Week 36>
Search Strategy:
----
1 Ebolavirus.od. or Ebola haemorrhagic fever/ (5197)
2 Marburg virus disease/ or Marburg marburgvirus/ (87)
3 Lassa virus/ or Lassa fever/ (1041)
4 Rift Valley fever virus.od. or Rift Valley fever.sh. (1981)
5 exp crimean-congo haemorrhagic fever virus/ (1856)
6 (ebola* or ebov or marburg* or lassa* or CCHF or CCHVF or "crimean-congo*" or "congo virus" or (crimean adj2 (hemorrhagic or haemorrhagic)) or "rift valley*" or RVF or RVFV).ti,ab. (11046)
7 exp severe acute respiratory syndrome-related coronavirus/ (86613)
8 Middle East respiratory syndrome coronavirus.od. (2140)
9 ("middle east* respiratory syndr*" or MERS-CoV or "novel CoV*" or "novel betacoronavirus" or coronavirus* or covid* or ("middle east" adj3 (cov or betacoronavirus*)) or (MERS adj3 (cov or betacoronavirus*)) or "mers-coronavirus" or "mers cov" or merscov or "wuhan flu" or 2019-nCoV).ti,ab. (89257)
10 West Nile fever.sh. or West Nile virus.od. (6612)
11 ("west nile" or "egypt 101").ti,ab. (7294)
12 influenza a virus subtype h1n1/ (7289)
13 influenza a/ (14712)
14 (H1N1 or H5N1 or H3N2 or H7N9).ti,ab. (16604)
15 exp henipavirus/ (1218)
16 (nipah* or hendra* or henipavirus*).ti,ab. (1218)
17 exp african swine fever virus/ (645)
18 ("african swine" or "ASF virus" or ASFV or "wart hog disease virus*").ti,ab. (589)
19 exp monkeypox virus/ (431)
20 (monkeypox or "monkey pox").ti,ab. (501)
21 exp zika virus/ (5511)
22 (zika* or zikv).ti,ab. (6974)
23 ("disease x*" or "pathogen x*").ti,ab. (169)
24 smallpox/ (1400)
25 (smallpox* or "small pox*" or variola).ti,ab. (2041)
26 exp human poliovirus 1/ or exp human poliovirus 2/ or exp human poliovirus 3/ (5089)
27 polio*.ti,ab. (7787)
28 brucellosis/ (6497)
29 (brucellosis or brucelloses or brucella).ti,ab. (8154)
30 q fever/ (3202)
31 ("q fever*" or "Coxiella burnetii" or coxiellosis or "query fever*").ti,ab. (4545)
32 melioidosis/ (1811)
33 (melioidosis or pseudomallei or "whitmore* disease*" or "nightcliff gardener* disease*" or pseudoglanders or "paddy-field disease" or "paddyfield disease").ti,ab. (2561)
34 exp francisella tularensis/ (2979)
35 (Tularaemia* or tularemia* or "Francisella tularensis" or "ohara disease" or "rabbit fever").ti,ab. (2849)
36 cholera/ (5792)

37 cholera*.ti,ab. (12380)
38 exp yersinia pestis/ (4823)
39 (plague or "black death" or "Yersinia pestis" or "Y. pestis").ti,ab. (5642)
40 exp anthrax/ (3450)
41 (Anthrax or "Bacillus anthracis" or "B. anthracis").ti,ab. (4632)
42 emerging infectious diseases/ (6986)
43 ("priority pathogen*" or "potential pathogen*" or "re-emerging pathogen*" or "reemerging pathogen*" or "emerging pathogen*").ti,ab. (5066)
44 outbreaks/ (52091)
45 exp epidemics/ (77111)
46 (((health$ or disease$) adj5 (disaster$ or catastrophe$ or crises or crisis)) or epidemic* or pandemic* or outbreak* or out-break* or "public health emergenc*" or "global health emergenc*").ti,ab. (182052)
47 1 or 2 or 3 or 4 or 5 or 6 or 7 or 8 or 9 or 10 or 11 or 12 or 13 or 14 or 15 or 16 or 17 or 18 or 19 or 20 or 21 or 22 or 23 or 24 or 25 or 26 or 27 or 28 or 29 or 30 or 31 or 32 or 33 or 34 or 35 or 36 or 37 or 38 or 39 or 40 or 41 or 42 or 43 or 44 or 45 or 46 (296286)
48 ((setting* adj2 priorit*) or (research* adj2 priorit*) or "research strateg*" or "prioriti?ation methodolog*" or (generat* adj2 priorit*) or (develop* adj2 priorit*) or "priority guideline*" or "agenda setting*" or (research* adj1 gap*) or (identif* adj2 priorit*) or "research question*").ti,ab. (11794)
49 (preparedness or readiness or "in advance of" or "planning for").ti,ab. (51741)
50 infection control/ (13058)
51 prevention/ (29143)
52 (response or control or prevention or addressing or tackle).ti,ab. (1023753)
53 49 or 50 or 51 or 52 (1067052)
54 47 and 48 and 53 (756)

SCOPUS

(((TITLE-ABS-KEY (ebola* OR ebov OR marburg* OR lassa* OR cchf OR cchvf OR "crimean-congo*" OR "congo virus" OR (crimean W/2 (hemorrhagic OR haemorrhagic)) OR "rift valley*" OR rvf OR rvfv) OR TITLE-ABS-KEY (("middle east* respiratory syndr*" OR mers-cov OR "novel CoV*" OR "novel betacoronavirus" OR coronavirus* OR covid* OR ("middle east" W/3 (cov OR betacoronavirus*)) OR (mers W/3 (cov OR betacoronavirus*)) OR "mers-coronavirus" OR "mers cov" OR merscov OR "wuhan flu" OR 2019-ncov)) OR TITLE-ABS-KEY ("west nile" OR "egypt 101" OR h1n1 OR h5n1 OR h3n2 OR h7n9 OR nipah* OR hendra* OR henipavirus* OR "african swine" OR "ASF virus" OR asfv OR "wart hog disease virus*" OR monkeypox OR "monkey pox" OR zika* OR zikv OR "disease x*" OR "pathogen x*") OR TITLE-ABS-KEY (smallpox* OR "small pox*" OR variola OR polio* OR brucellosis OR brucelloses OR brucella OR "q fever*" OR "Coxiella burnetii" OR coxiellosis OR "query fever*" OR melioidosis OR pseudomallei OR "whitmore* disease*" OR "nightcliff gardener* disease*" OR pseudoglanders OR "paddy-field disease" OR "paddyfield disease" OR tularaemia* OR tularemia* OR "Francisella tularensis" OR "ohara disease" OR "rabbit fever" OR cholera* OR plague OR "black death" OR "Yersinia pestis" OR "Y. pestis" OR anthrax OR "Bacillus anthracis" OR "B. anthracis"))) OR ((TITLE-ABS-KEY ("priority pathogen*" OR "potential pathogen*" OR "re-emerging pathogen*" OR "reemerging pathogen*" OR "emerging pathogen*") OR TITLE-ABS-KEY (((((health$ OR disease$) W/5 (disaster$ OR catastrophe$ OR crises OR crisis)) OR epidemic* OR pandemic* OR outbreak* OR out-break* OR "public health emergenc*" OR "global health emergenc*"))))) AND (TITLE-ABS-KEY (((setting* W/2 priorit*) OR (research* W/2 priorit*) OR "research strateg*" OR "prioriti?ation methodolog*" OR (generat* W/2 priorit*) OR (develop* W/2 priorit*) OR "priority guideline*" OR "agenda setting*" OR (research* W/1 gap*) OR (identif* W/2 priorit*) OR "research question*"))) AND ((TITLE-

ABS-KEY (preparedness OR readiness OR "in advance of" OR "planning for") OR TITLE-ABS-KEY (response OR control OR prevention OR addressing OR tackle)))

WHO Global Index Medicus https://pesquisa.bvsalud.org/gim/
(tw:(ebola* OR ebov OR marburg* OR lassa* OR cchf OR cchvf OR "crimean-congo*" OR "congo virus" OR (crimean AND (hemorrhagic OR haemorrhagic)) OR "rift valley*" OR rvf OR rvfv or "middle east* respiratory syndr*" OR mers-cov OR "novel CoV*" OR "novel betacoronavirus" OR coronavirus* OR covid* OR ("middle east" AND (cov OR betacoronavirus*)) OR (mers AND (cov OR betacoronavirus*)) OR "mers-coronavirus" OR "mers cov" OR merscov OR "wuhan flu" OR 2019-ncov OR "west nile" OR "egypt 101" OR h1n1 OR h5n1 OR h3n2 OR h7n9 OR nipah* OR hendra* OR henipavirus* OR "african swine" OR "ASF virus" OR asfv OR "wart hog disease virus*" OR monkeypox OR "monkey pox" OR zika* OR zikv OR "disease x*" OR "pathogen x*" OR smallpox* OR "small pox*" OR variola OR polio* OR brucellosis OR brucelloses OR brucella OR "q fever*" OR "Coxiella burnetii" OR coxiellosis OR "query fever*" OR melioidosis OR pseudomallei OR "whitmore* disease*" OR "nightcliff gardener* disease*" OR pseudoglanders OR "paddy-field disease" OR "paddyfield disease" OR tularaemia* OR tularemia* OR "Francisella tularensis" OR "ohara disease" OR "rabbit fever" OR cholera* OR plague OR "black death" OR "Yersinia pestis" OR "Y. pestis" OR anthrax OR "Bacillus anthracis" OR "B. anthracis" OR "priority pathogen*" OR "potential pathogen*" OR "re-emerging pathogen*" OR "reemerging pathogen*" OR "emerging pathogen*" OR ((health* OR disease*) AND (disaster* OR catastrophe* OR crises OR crisis)) OR epidemic* OR pandemic* OR outbreak* OR out-break* OR "public health emergenc*" OR "global health emergenc*")) AND (tw:((setting* AND priorit*) OR (research* AND priorit*) OR "research strateg*" OR "prioritisation methodolog*" OR "prioritization methodolog*" OR (generat* AND priorit*) OR (develop* AND priorit*) OR "priority guideline*" OR "agenda setting*" OR (research* AND gap*) OR (identif* AND priorit*) OR "research question*")) AND (tw:(preparedness OR readiness OR "in advance of" OR "planning for" or response OR control OR prevention OR addressing OR tackle))

Google Scholar
(priorit*|"agenda setting"|"research gap*"|"research question*")(preparedness|readiness|"advance of"|"planning for"|response|control|prevention|addressing|tackle)(outbreak*|pandemic*|epidemic*|"public health emergenc*"|"global health emerg*")

WHO websites
https://www.google.com/search?hl=en&as_q=priority&as_epq=outbreak*&as_oq=preparedness+readiness+response+%22advance+of%22+%22planning+for%22+control+prevention+addressing&as_eq=&as_nlo=&as_nhi=&lr=&cr=&as_qdr=all&as_sitesearch=.who.int&as_occt=any&safe=images&as_filetype=&tbs=

priority preparedness OR readiness OR response OR "advance of" OR "planning for" OR control OR prevention OR addressing "outbreak*" site:.who.int

https://www.google.com/search?q=priority+preparedness+OR+readiness+OR+response+OR+%22advance+of%22+OR+%22planning+for%22+OR+control+OR+prevention+OR+addressing+%22outbreak*%22+site%3A.paho.org&lr=&safe=images&hl=en&as_qdr=all&ei=CZVCZLSOHrWYhbIP35C0yA8&ved=0ahUKEwj0toC1j7v-AhU1TEEAHV8IDfkQ4dUDCA8&uact=5&oq=priority+preparedness+OR+readiness+OR+response+OR+%22advance+of%22+OR+%22planning+for%22+OR+control+OR+prevention+OR+addressing+%22outbreak*%22+site%3A.paho.org&gs_lcp=Cgxnd3Mtd2l6LXNlcnAQAzITCAAQ

jwEQ6gIQtAIQjAMQ5QIYATITCAAQjwEQ6gIQtAIQjAMQ5QIYATITCAAQjwEQ6gIQtAIQjAMQ5QIYATITCAAQjwEQ6gIQtAIQjAMQ5QIYATITCAAQjwEQ6gIQtAIQjAMQ5QIYATITCAAQjwEQ6gIQtAIQjAMQ5QIYATITCC4QjwEQ6gIQtAIQjAMQ5QIYATITCC4QjwEQ6gIQtAIQjAMQ5QIYATITCAAQjwEQ6gIQtAIQjAMQ5QIYATITCAAQjwEQ6gIQtAIQjAMQ5QIYAUoECEEYAFAAWNbDAWDRxQFoAnAAeACAAQCIAQCSAQCYAQCgAQGgAQKwAQrAAQHaAQQIARgK&sclient=gws-wiz-serp

priority preparedness OR readiness OR response OR "advance of" OR "planning for" OR control OR prevention OR addressing "outbreak*" site:.paho.org

2. Data Extraction Table for Scoping Review

Field	Description	Details
**1. Map and describe research prioritisation exercises for high consequence pathogens**		
Title	Title of the article	Title under which the resource was published
Date of publication	Date of publication as documented	Exact date on which the resources was published – year only if exact date not available
Type of publication	Publication type as documented	E.g. policy paper, journal article, reports, ...
Type of article	Article type as documented	E.g. research article, commentary, review, opinion piece...
Author	All named authors as documented	All named authors documented
Version of research agenda	Version of research agenda as documented	E.g. version 1, version 2, ...
Duration of research agenda	Duration of validity of research priorities	E.g. 1 year, 4 months, range of years (XXXX-XXXX), ...
Pathogen(s) of interest	Indicate the pathogen(s) that are addressed in the article	E.g. COVID-19, Ebola, Influenza, ...
Purpose	The main purpose of the article	Preparedness, response or both
Key focus	Key discipline area of focus	E.g. vaccine research for COVID-19, mental health research for Ebola, ...
Geographical target	Country/ies or region(s) to which the priority setting is designed for	National, regional, global, other
		If “other” describe level
		Details of countries and regions
Target population	Indicate the populations targeted where available	E.g. women, children, indigenous populations, ...
Broad priority areas	An outline of the broad priorities set	E.g. epidemiological study, vaccine research, ...

**2. Identify and describe the approaches used in priority setting for high consequence pathogens**		
Named methodology employed	Indicate name of method where available	E.g., CAM 3D, CHNRI, COHRED, ...
Type of methodology employed	Type and count of method employed for prioritisation	E.g. interview, meetings, surveys, reviews, ...
		Consultation: Yes, No
		Database review: Yes, No
		Literature synthesis: Yes, No
		Number of modalities (1, 2, ...)
Duration of priority setting process	Duration as reported	E.g. 2 months, 3 years, ...
Criteria used to group the priorities	Criteria described in grouping priorities	Emergent, pre-determined, unspecified
Method used to group the priorities	Methods described in grouping priorities	E.g. voting, ranking, ...
Criteria for shortlisting priorities	Criteria used for selecting priorities as documented	E.g. need, feasibility, equity, ...
Mode of consultation	Mode of consultation as described	In-person, online, unspecified
Stakeholders/contributors	Participants involved in the priority setting if there were deliberations/surveys/meeting	E.g. patients, clinicians, communities, policymakers, funders, experts, ...
Communication plan	An outline of strategies to be used in disseminating the set priorities	Yes, no
		E.g. publications, press release, website launch, ...
**3. Identify and describe monitoring and evaluation activities on progress for prioritised research areas**		
Processes for M&E	An outline of key M&E activities planned/undertaken for the set priorities	Yes, no
		Description of the M&E planned activities
Review period	Indicate planned timeline for review	E.g. annual, 5-year period, ...
Responsible entities	Who is responsible for the M&E and any regulations for that/any plans for M&E	Indicate the responsibility as stated
**4. Describe research priorities**		
Categories/levels/layers of priorities	Describe how the priorities were further sub-grouped under each of the broad priorities	Include the details of the broad priority areas included
WHO Roadmap priority areas	Map research priorities to the adapted WHO research roadmap for COVID-19	Indicate which WHO roadmap area(s) the priorities map to

Acronyms - CAM 3D: Combined Approach Matrix 3D, CHNRI: Child Health and Nutrition Research Initiative, COHRED: Council on Health Research for Development.

3. Characteristics of the included publications

First Author (name and publication year)	Pathogen of article focus	Key focus of document
Kemp (2021)[1]	SARS-CoV-2	Research priorities for biological security in the United Kingdom to address technological advances and trends in globalisation
Akomea-Frimpong (2022)[2]	SARS-CoV-2	Impact of COVID-19 on public–private partnership (PPP) arrangements in the construction industry
Bucher (2022)[3]	SARS-CoV-2	Track progress on the WHO COVID-19 research priorities, LMIC priorities and UN Recovery Roadmap priorities
Bucher (2021)[4]	SARS-CoV-2	Track progress on the WHO COVID-19 research priorities, LMIC priorities and UN Recovery Roadmap priorities
Bucher (2021)[5]	SARS-CoV-2	Track progress on the WHO COVID-19 research priorities and LMIC priorities
Bucher (2022)[6]	SARS-CoV-2	Track progress on the WHO COVID-19 research priorities, LMIC priorities and UN Recovery Roadmap priorities
Bucher (2021)[7]	SARS-CoV-2	Track progress on the WHO COVID-19 research priorities and LMIC priorities and priorities
Bucher (2020)[8]	SARS-CoV-2	Track progress on the WHO COVID-19 research priorities, LMIC priorities and priorities
Sigfrid (2019)[9]	Lassa Virus	Identify existing evidence gaps across pre-defined clinical domains of Lassa fever within a limited period of time for rapid response
Moore (2021)[10]	Epidemic/Pandemic Influenza viruses	Identify major issues and priority areas in the Influenza Vaccines Research and Development (R&D) Roadmap (IVR) to address these
Majid (2021)[11]	SARS-CoV-2	Perform a systematic mapping exercise of non-clinical evidence syntheses pertaining to Covid-19 to help generate evidence-based recommendations for governments worldwide

Harris (2022)[12]	SARS-CoV-2	Provide an evidenced-based framework for priority clinical research in the current COVID-19 outbreak and highlight the research gaps, and provide recommendations for the implementation of standardised protocols
Ko (2022)[13]	Vibrio cholerae	Provide additional evidence to assist countries in controlling cholera and provide a prioritized list of research questions to focus on for the implementation of the Ending Cholera Global Roadmap
Wiwanitkit (2015)[14]	Ebola virus	Promote research on a combined active and passive system to combat the present extending Ebola outbreak
Norton (2020)[15]	SARS-CoV-2	Track progress on the WHO COVID-19 research priorities and LMIC priorities
Ng (2020)[16]	SARS-CoV-2	Provide future research questions for breastfeeding and infant care when mother is infected with COVID-19
WHO (1980)[17]	Vibrio cholerae	Review the important new information, and identifies gaps in our knowledge, on cholera and other vibrio-associated diarrhoeas
Mechanick (2021)[18]	SARS-CoV-2	Examine nutrition research applicable to the COVID-19 pandemic and the research/knowledge gaps.
Al-Riyami (2021)[19]	SARS-CoV-2	Aims to identify existing key gaps in current knowledge in the clinical application of COVID-19 convalescent plasma, notably on its dosing, safety and effectiveness.
Goldenberg (2020)[20]	SARS-CoV-2	Provide consensus-based recommendations on the use of anticoagulant thromboprophylaxis in children hospitalized for COVID-19-related illnesses, and identify priorities for future research.
Bueno de Mesquita (2021)[21]	SARS-CoV-2	Review evidence of indoor environmental controls against transmission and identify need for investments in research
Mody (2022)[22]	SARS-CoV-2	Identifies knowledge gaps and challenges in healthcare epidemiology research related to coronavirus disease 2019 (COVID-19) with a focus on core principles of healthcare epidemiology.
Liu (2020)[23]	SARS-CoV-2	Analysis of early COVID-19 research to inform research prioritisation and policy planning both in

		the current COVID-19 pandemic and similar global health crises
Dudovitz (2021)[24]	SARS-CoV-2	Develop a research agenda to understand the short- and long-term mechanisms and impacts of the COVID-19 pandemic on children's healthy development
Rylett (2020)[25]	SARS-CoV-2	Priority areas for research on the impact of the Covid-19 pandemic on older adults that have been identified by the CIHR Institute of Aging
Lindsley (2020)[26]	SARS-CoV-2	Present some of the questions surrounding aerosols containing SARS-CoV-2 and to provide suggestions for future research topics, especially on spread and effectiveness of protective measures
Bonney (2020)[27]	SARS-CoV-2	Propose ten research questions that when answered in a timely manner by scientists in Africa, will enhance Africa's response to the pandemic
Semenza (2021)[28]	SARS-CoV-2	Conduct an assessment of research gaps that can help inform policy decisions regarding the COVID-19 response
Shook (2021)[29]	SARS-CoV-2	Summarize the literature to date on COVID-19 vaccination in pregnancy and lactation and highlight opportunities for investigation that may inform future maternal vaccine development and implementation strategies
de la Rica (2020)[30]	SARS-CoV-2	Summarize the latest data available about the role of the cytokine storm in COVID-19 disease severity as well as potential therapeutic approaches to ameliorate it, identify gaps in our knowledge and suggest priorities for future research aimed at stratifying patients according to risk as well as personalizing therapies in the context of COVID19-driven hyperinflammation
Liu (2021)[31]	SARS-CoV-2	Aim to describe the impact of the pandemic on dementia wellbeing and identify priorities for future research
Errett (2020)[32]	SARS-CoV-2	Identify and prioritize environmental health sciences Severe Acute Respiratory Syndrome Coronavirus 2 (SARS-CoV-2) and associated Coronavirus Disease 2019 (COVID-19) research questions.

Uyeki (2016)[33]	MERS-CoV	Identify medical countermeasures for MERS-CoV
Payne (2007)[34]	Bacillus anthracis	Describe the process by which the Vaccine Analytic Unit's anthrax vaccine safety research plan was developed following a comprehensive review of these topics and recommend topics as potentially warranting further study
Poland (2018)[35]	Zika virus	Examine vaccine development efforts for Zika virus to date and research gaps in the development of candidate vaccines against Zika virus.
GloPID-R (2020)[36]	SARS-CoV-2	Establish collaboration and identify knowledge gaps, in order to build a collective path forward to end COVID-19
Cowling (2020)[37]	SARS-CoV-2	Improve information on epidemiology and countermeasures in order to calibrate public health responses
Evans (2021)[38]	SARS-CoV-2	Identify gaps in the current evidence base and identify research priorities in the local maternity setting context during the Covid-19 pandemic
Clemens (2011)[39]	Vibrio cholerae	Provision of evidence addressing evidence on the performance of enteric vaccines will help expand the use of enteric vaccines in developing countries
European Food Safety Authority (2015)[40]	Epidemic/Pandemic Influenza viruses	Continuous priority research gap analysis for animal influenza
Hand (2021)[41]	SARS-CoV-2	Share gaps in knowledge and research related to pandemic management identified by nurse leaders during the COVID-19 pandemic
Destoumieux-Garzón (2022)[42]	SARS-CoV-2	Propose long-term research questions regarding COVID-19 and emerging infectious diseases (EIDs) that are based on effective integration of environmental, ecological, evolutionary, and social sciences to better anticipate and mitigate EIDs
Vearey (2021)[43]	SARS-CoV-2	Explore responses to COVID-19 to provide an improved understanding of the politics influencing the entanglement between global health, immigration governance, and the global health security during epidemics. Catalyse a new and evolving research agenda to inform the development and implementation of appropriate

		pandemic responses in a region associated with some of the highest levels of inequality globally
Abu-Raya (2021)[44]	SARS-CoV-2	Provide a review of the current literature on COVID-19 vaccines in pregnant women, identifies knowledge gaps and outlines priorities for future research to optimize protection against SARS-CoV-2 in the pregnant women and their infants
Etti (2021)[45]	SARS-CoV-2	Conduct an international survey to identify global research priorities for COVID-19 in maternal, reproductive and child health
Centers for Disease Control and Prevention (2000)[46]	West Nile Virus	Review the outbreak of WNV and to provide input and guidance on the programs that should be developed to monitor WN virus activity and to prevent future outbreaks of disease
Baral (2021)[47]	SARS-CoV-2	Inform the development of the November 2020 United Nations Research Roadmap for the COVID-19 Recovery, by providing a synthesis of available evidence on the impact of pandemics and epidemics on (1) essential services and (2) health systems preparedness and strengthening
Simpson (2020)[48]	monkeypox virus	Review the status of human monkeypox disease, highlighting facts and deficient understanding as well as research gaps
Vallès (2020)[49]	Yersinia pestis	Research priorities to plague outbreaks " framed in One health approach"
Siegfried (2017)[50]	Other	Assessment conducted to identify perceived knowledge gaps, information needs, and research priorities among state, territorial, and local public health preparedness directors and coordinators related to public health emergency preparedness and response (PHPR)
Zhang (2020)[51]	SARS-CoV-2	Analysis of research trends related to coronavirus over 20 years to point out the highlights and identify gap areas
McMahon (2020)[52]	SARS-CoV-2	Conduct a rapid-cycle priority identification process to inform Canada’s Health System Response to COVID-19 on priorities for Health Services and Policy Research (HSPR)
Rojek (2017)[53]	Ebola virus	Discuss new insights from patient-oriented research completed during the west Africa epidemic, identify ongoing knowledge gaps, and suggest priorities for future research

Corbel (2017)[54]	Other	Identify strategies for the development and implementation of standardized insecticide resistance management, also to allow comparisons across nations and across time, and to define research priorities for control of vectors of arboviruses
Noel (2020)[55]	SARS-CoV-2	Identify key knowledge gaps for COVID-19 in children and to fill those gaps to best address the needs of children, especially in clinical settings
Yazdizadeh (2022)[56]	SARS-CoV-2	Identify the knowledge gaps and suggest research priorities in response to the COVID-19 epidemic in Iran
Jacobsen (2016)[57]	Ebola virus	Reflect on how to improve the detection of and coordinated response to future epidemics based on the lessons learned from the Ebola epidemic
Sutton (2021)[58]	SARS-CoV-2	Identify research questions that are fundamental to the communication challenges that have emerged under the threat of COVID-19 to offer guidance to scholars engaging in practitioner-informed research and provides risk communicators with a set of research questions to guide future knowledge needs
Dao (2022)[59]	SARS-CoV-2	Scoping review to map publications on COVID-19 in Vietnam in order to guide research priorities and policies in the country
Grace (2012)[60]	Other	Present data and expert knowledge on poverty and zoonoses hotspots to inform prioritisation of study areas on the transmission of disease in emerging livestock systems in the developing world
Aguannoa (2018)[61]	MERS-Cov	Summarize progress in research on Middle East Respiratory Syndrome since a FAO-OIE-WHO Global Technical Meeting
Memish (2020)[62]	MERS-CoV	Update on the current knowledge and perspectives on MERS epidemiology, virology, mode of transmission, pathogenesis, diagnosis, clinical features, management, infection control, development of new therapeutics and vaccines, and highlights unanswered questions and priorities for research, improved management, and prevention
Malik (2016)[63]	MERS-CoV	Summarize current understanding and gaps in knowledge about Middle East respiratory syndrome coronavirus (MERS-CoV), including its

		origin, transmission, effective control measures and management
Shrivastava (2020)[64]	SARS-CoV-2	Research priorities for a better public health response to the COVID-19 pandemic
Khan (2008)[65]	Lassa Virus	Review the present literature on treatment and pathogenesis of Lassa Fever and outline priorities for future research in the field
Weerakkody (2022)[66]	SARS-CoV-2	To answer questions about the utility, safety, and outcome benefit of NIRS strategies and to outline research priorities to guide further clinical decision making
Gupta (2020)[67]	SARS-CoV-2	Study research trends, explore research gaps, and provide directions for more efficient and effective research in the future for operations management research
Faghy (2022)[68]	SARS-CoV-2	Set out a series of research priorities that could inform interdisciplinary collaboration
Lentine (2021)[69]	SARS-CoV-2	Reflect on the practice decisions made by the transplant community in the initial response to the pandemic, consider lessons learned, and discuss the areas in which we are still practicing with uncertainty, and look ahead to the next phase of the pandemic response
Norton (2021)[70]	SARS-CoV-2	Outline priorities for COVID-19 preparedness and response in low-resource settings
Manning (2021)[71]	SARS-CoV-2	Identify priorities for research in relation to the COVID-19 pandemic and 'beyond', as recommended by nurses, midwives and health visitors
Ault (2012)[72]	Epidemic/Pandemic Influenza viruses	Review programmatic and research priorities with regard to overcoming barriers to influenza immunization of pregnant women
Cassetti (2022)[73]	Other	Leveraging a prototype pathogen approach in biomedical research preparedness and response
Azim (2022)[74]	SARS-CoV-2	Identify gaps in knowledge and prioritize a research agenda that is linked to public health action in the WHO South-East Asia Region

Highet (2021)[75]	SARS-CoV-2	Provide a robust synthesis of key concepts and existing evidence to support the identification and appraisal of research priorities for social protection and basic services
Papa (2015)[76]	Crimean-Congo Haemorrhagic Fever virus	Advances in research including eco-epidemiology, basic virology, clinical virology, prophylaxis and therapy of Crimean-Congo haemorrhagic fever (CCHF)
Yassi (2005)[77]	SARS-CoV-1	Preventing occupational respiratory disease transmission in HCWs
Folayan (2018)[78]	Other	Community's perspectives on types of research prioritised in outbreak
Simpson (2021)[79]	Other	Strength and weakness in the control of zoonoses
Tong (2021)[80]	SARS-CoV-2	Sensor technology for COVID-19
Programme for Control of Diarrhoeal Diseases (1991)[81]	Vibrio cholerae	Vaccine development for diarrhoeal diseases caused by Rotaviruses, Shigella, Vibrio cholerae, and enterotoxigenic Escherichia coli (ETEC)
Wiwanitkit (2013)[82]	Epidemic/Pandemic Influenza viruses	Modelling the transmission risks (bird to human and human to human) of H7N9 bird flu
French (2009)[83]	Epidemic/Pandemic Influenza viruses	Pandemic influenza response in Canada and knowledge gaps
Board on Health Sciences Policy (2014)[84]	Ebola virus	Research priorities to inform public health and medical practice for EBV on transmission, and training of PPE usage for of traditional workers
Polašek (2022)[85]	SARS-CoV-2	Priorities for health research that would have the potential to reduce the impact of the COVID-19 pandemic in LMICs.
Nederman (2020)[86]	SARS-CoV-2	COVID-19 research for pulmonary and critical care

Reese (2020)[87]	SARS-CoV-2	How COVID-19 influences environmental psychology e.g. future responses to climate change
Eder (2018)[88]	Other	Research priorities for transmission dynamics, vectorial capacity, and co-infection of vector borne disease in urban settings
Mehta (2021)[89]	SARS-CoV-2	Public health research in maternal and child health priorities for COVID-19 in India
Khazaie (2020)[90]	SARS-CoV-2	Social and behavioural sciences response to inform knowledge and management of the pandemic
Otmani del Barrio (2018)[91]	Other	Prevention and control of vector born and other infectious diseases of poverty in urban populations
Milone (2020)[92]	SARS-CoV-2	Shortcomings in resources and surgical challenges during the COVID-19 pandemic
Johnson (1975)[93]	Lassa Virus	Research priorities for future research on the arenaviruses based on Lassa virus
Rizzoli (2015)[94]	West Nile Virus	Challenge of West Nile Virus in Europe
Barouki (2020)[95]	SARS-CoV-2	Connections between COVID-19 and the Environment, Climate and Health nexus in the EU
Sharp (2021)[96]	SARS-CoV-2	Evaluate existing scientific literature on menstrual cycle feature changes in the COVID-19 pandemic
Agarwal (2020)[97]	Zika virus	Zika virus history, epidemiology, evolution, transmission, pathogenesis, clinical signs and unusual presentations, laboratory diagnosis, treatment, prevention and control
Blasiak (2022)[98]	SARS-CoV-2	AI-based platform to identify candidate therapies for COVID-19
Gupta (2020)[99]	SARS-CoV-2	Knowledge Gaps in Research priorities for the COVID-19 outbreak management

Norton (2020)[100]	SARS-CoV-2	Global perspective of COVID-19 research priorities built on WHO roadmap
Calnan (2017)[101]	Ebola virus	Research needs and priorities from the perspective of people who directly affected by EVD epidemic in Guinea
Jarman (2020)[102]	SARS-CoV-2	Surgical outcome and health services during Covid-19 pandemic
Coopersmith (2021)[103]	SARS-CoV-2	Management, pathophysiology, and host response of critically ill COVID-19 patients
Benowitz (2022)[104]	SARS-CoV-2	Tobacco product use to SARS-CoV-2 infection and COVID-19 outcomes, and research priorities for acute and post-acute health outcomes of COVID-19
Sinha (2020)[105]	SARS-CoV-2	Research and programming priorities towards post-COVID-19 Mental Health Friendly Cities
Bausch (2008)[106]	Ebola virus, Marburg virus	Research priorities and environment for field study of the filovirus haemorrhagic fevers and a strategy for future prospective clinical research on treatment and vaccine prevention
Stegenga (2021)[107]	SARS-CoV-2	Research questions/agendas for epidemic diseases by medical research organisations
Wikan (2016)[108]	Zika virus	Transmission; virological; immunological considerations of Zika virus in South-East Asia
Wilder-Smith (2017)[109]	Other	Research priorities and public health interventions for aedes-transmitted diseases
Cowling (2015)[110]	Ebola virus	Research needs in Ebola outbreak
Abela-Ridder (2010)[111]	Epidemic/Pandemic Influenza viruses	Scientific aspects of the zoonotic and pandemic threats posed by H5N1 and other zoonotic influenza viruses
Smith (2006)[112]	SARS-CoV-1	The role that risk, and especially the perception of risk, played in driving the economic impact of SARS

WHO (2020)[113]	SARS-CoV-2	To coordinate and accelerate the development of research and innovations to control the epidemic of threatening diseases
African Academy of Sciences (2020)[114]	SARS-CoV-2	Prioritisation list for research and development for the COVID-19 outbreak in Africa
Africa CDC (2021)[115]	SARS-CoV-2	Research and development priorities for COVID-19 in Africa
United Nations (2020)[116]	SARS-CoV-2	Long covid clinical characteristics
Carson (2021)[117]	SARS-CoV-2	Long covid clinical characteristics
Paparella (2021)[118]	SARS-CoV-2	Possible future scenarios to frame the uncertainty around COVID-19 and infectious diseases and provide research recommendations
Clarke (2021)[119]	SARS-CoV-2	Research agenda on Long COVID's impact on patients, carers and healthcare professionals
WHO (2022)[120]	SARS-CoV-2	Global research agenda and innovation for COVID-19 and pandemic preparedness and response
US DHHS (2022)[121]	SARS-CoV-2	Research action plan on prevention, diagnosis, treatment and service provision for individuals and families experiencing Long COVID
PAHO/ WHO (2016)[122]	Zika virus	Research agenda for characterizing the Zika virus outbreak and actions to limit the outbreak's impact
WHO (2022)[123]	monkeypox virus	Guide for coordinated public health actions to stop monkeypox outbreak
WHO (2021)[124]	SARS-CoV-2	Progress, gaps and research priorities for community-centred approaches to health emergencies
WHO (2017)[125]	Zika virus	Reviewing evidence on sexual transmission of ZIKV, the research gaps, and study methods

The data generated and analysed during the current study are available in Figshare - https://doi.org/10.25446/oxford.c.7235395

4. References of included studies

1. Kemp L, Aldridge DC, Booy O, Bower H, Browne D, Burgmann M, et al. 80 questions for UK biological security. PLoS One [Internet]. 2021;16(1). Available from: https://www.scopus.com/inward/record.uri?eid=2-s2.0-85099440450&doi=10.1371%2Fjournal.pone.0241190&partnerID=40&md5=7fba0bea7241c0aea7b72aa3d2cc1a8a

2. Akomea-Frimpong I, Jin X, Osei-Kyei R, Tumpa RJ. A critical review of public–private partnerships in the COVID-19 pandemic: key themes and future research agenda. Smart and Sustainable Built Environment [Internet]. 2022; Available from: https://www.scopus.com/inward/record.uri?eid=2-s2.0-85129263422&doi=10.1108%2FSASBE-01-2022-0009&partnerID=40&md5=4a571385181db4da35c2fbb92a0cf0b3

3. Norton A, Bucher A, Antonio E, Grund H, Jabin N, Jones C, et al. A living mapping review for COVID-19 funded research projects: 15 month update. Wellcome Open Res. 2022;5.

4. Bucher A, Antonio E, Grund H, Jabin N, Jones C, Kifle M, et al. A living mapping review for COVID-19 funded research projects: 18 month update. Wellcome Open Res [Internet]. 2020;5:209. Available from: http://ovidsp.ovid.com/ovidweb.cgi?T=JS&PAGE=reference&D=pmnm&NEWS=N&AN=33117894

5. Norton A, Bucher A, Antonio E, Advani N, Grund H, Mburu S, et al. A living mapping review for COVID-19 funded research projects: nine-month update. Wellcome Open Res [Internet]. 2020;5:209. Available from: http://ovidsp.ovid.com/ovidweb.cgi?T=JS&PAGE=reference&D=medv&NEWS=N&AN=33117894

6. Norton A, Bucher A, Antonio E, Advani N, Johnston C, Grund H, et al. A living mapping review for COVID-19 funded research projects: one year update. Wellcome Open Res [Internet]. 2020;5:209. Available from: http://ovidsp.ovid.com/ovidweb.cgi?T=JS&PAGE=reference&D=medv&NEWS=N&AN=33117894

7. Norton A, Bucher A, Antonio E, Advani N, Grund H, Mburu S, et al. A living mapping review for COVID-19 funded research projects: six-month update. Wellcome Open Res [Internet]. 2020;5:209. Available from: http://ovidsp.ovid.com/ovidweb.cgi?T=JS&PAGE=reference&D=medv&NEWS=N&AN=33117894

8. Norton A, Bucher A, Antonio E, Advani N, Grund H, Mburu S, et al. A living mapping review for COVID-19 funded research projects: three-month update. Wellcome Open Res [Internet]. 2020;5:209. Available from: http://ovidsp.ovid.com/ovidweb.cgi?T=JS&PAGE=reference&D=medv&NEWS=N&AN=33117894

9. Sigfrid L, Moore C, Salam AP, Maayan N, Hamel C, Garritty C, et al. A rapid research needs appraisal methodology to identify evidence gaps to inform clinical research priorities in response to outbreaks - Results from the Lassa fever pilot. BMC Med [Internet]. 2019;17(1). Available from: https://www.scopus.com/inward/record.uri?eid=2-s2.0-85067131976&doi=10.1186%2Fs12916-019-1338-1&partnerID=40&md5=8d20b4d2ca8849cac7860991b48435cf

10. Moore KA, Ostrowsky JT, Kraigsley AM, Mehr AJ, Bresee JS, Friede MH, et al. A Research and Development (R&D) roadmap for influenza vaccines: Looking toward the future. Vaccine [Internet]. 2021;39(45):6573–84. Available from: https://www.scopus.com/inward/record.uri?eid=2-s2.0-85117200775&doi=10.1016%2Fj.vaccine.2021.08.010&partnerID=40&md5=f7977c827a7c013446dbd077b9669db8

11. Majid U, Hussain SAS, Wasim A, Farhana N, Saadat P. A Systematic Map of Non-Clinical Evidence Syntheses Published Globally on COVID-19. Disaster Med Public Health Prep [Internet]. 2021; Available from: https://www.scopus.com/inward/record.uri?eid=2-s2.0-85111099482&doi=10.1017%2Fdmp.2021.236&partnerID=40&md5=e31efe183d322efd09f53d20e0384725

12. Harris C, Carson G, Baillie JK, Horby P, Nair H. An evidence-based framework for priority clinical research questions for COVID-19. J Glob Health [Internet]. 2020;10(1):11001. Available from: http://ovidsp.ovid.com/ovidweb.cgi?T=JS&PAGE=reference&D=emed21&NEWS=N&AN=631442309

13. Ko M, Cherian T, Groves HT, Klemm EJ, Qazi S. Application of the Child Health and Nutrition Research Initiative (CHNRI) methodology to prioritize research to enable the implementation of Ending Cholera: A global roadmap to 2030. PLoS One [Internet]. 2022;17(5). Available from: https://www.scopus.com/inward/record.uri?eid=2-s2.0-85130795466&doi=10.1371%2Fjournal.pone.0264952&partnerID=40&md5=36b800ce9aac9bf68a301f4b05343c26

14. Wiwanitkit V, Tambo E, Ugwu EC, Ngogang JY, Zhou XN. Are surveillance response systems enough to effectively combat and contain the Ebola outbreak? Infect Dis Poverty [Internet]. 2015;4(1). Available from: https://www.scopus.com/inward/record.uri?eid=2-s2.0-85006191478&doi=10.1186%2F2049-9957-4-7&partnerID=40&md5=c19940642d4324206bacb1a8bdbae0a3

15. Norton A, Bucher A, Antonio E, Advani N, Grund H, Mburu S, et al. Baseline results of a living systematic review for COVID-19 funded research projects. Wellcome Open Res [Internet]. 2020;5. Available from: https://www.scopus.com/inward/record.uri?eid=2-s2.0-

85096398667&doi=10.12688%2Fwellcomeopenres.16259.1&partnerID=40&md5=ca3b9d31f14e56e68ceb21fc9ed544af

16. Ng YPM, Low YF, Goh XL, Fok D, Amin Z. Breastfeeding in COVID-19: A Pragmatic Approach. Am J Perinatol [Internet]. 2020;37(13):1377–84. Available from: https://www.scopus.com/inward/record.uri?eid=2-s2.0-85091829043&doi=10.1055%2Fs-0040-1716506&partnerID=40&md5=78e40154e11435fa0457d501ba6375e4

17. World Health Organization (WHO). Cholera and other vibrio-associated diarrhoeas. Bull World Health Organ [Internet]. 1980;58(3):353–74. Available from: https://www.scopus.com/inward/record.uri?eid=2-s2.0-0019217443&partnerID=40&md5=141059d6e050c1f1deaaa66fdc8aa241

18. Mechanick JI, Carbone S, Dickerson RN, Hernandez BJD, Hurt RT, Irving SY, et al. Clinical Nutrition Research and the COVID-19 Pandemic: A Scoping Review of the ASPEN COVID-19 Task Force on Nutrition Research. Journal of Parenteral and Enteral Nutrition [Internet]. 2021;45(1):13–31. Available from: https://www.scopus.com/inward/record.uri?eid=2-s2.0-85096683369&doi=10.1002%2Fjpen.2036&partnerID=40&md5=1a7bbb5742f08a5fba4a047b93ca556c

19. Al-Riyami AZ, Schäfer R, van den Berg K, Bloch EM, Estcourt LJ, Goel R, et al. Clinical use of Convalescent Plasma in the COVID-19 pandemic: a transfusion-focussed gap analysis with recommendations for future research priorities. Vox Sang [Internet]. 2021;116(1):88–98. Available from: https://www.scopus.com/inward/record.uri?eid=2-s2.0-85087288683&doi=10.1111%2Fvox.12973&partnerID=40&md5=fa2211cf86149cb5a329569e20f7abf1

20. Goldenberg NA, Sochet A, Albisetti M, Biss T, Bonduel M, Jaffray J, et al. Consensus-based clinical recommendations and research priorities for anticoagulant thromboprophylaxis in children hospitalized for COVID-19–related illness. Journal of Thrombosis and Haemostasis [Internet]. 2020;18(11):3099–105. Available from: https://www.scopus.com/inward/record.uri?eid=2-s2.0-85094869393&doi=10.1111%2Fjth.15073&partnerID=40&md5=d468c724aca37e9fa35f076228f4abc4

21. Bueno de Mesquita PJ, Delp WW, Chan WR, Bahnfleth WP, Singer BC. Control of airborne infectious disease in buildings: Evidence and research priorities. Indoor Air [Internet]. 2022;32(1). Available from: https://www.scopus.com/inward/record.uri?eid=2-s2.0-85119653772&doi=10.1111%2Fina.12965&partnerID=40&md5=16d07ebc687ff6fdc14aad04c129976f

22. Mody L, Akinboyo IC, Babcock HM, Bischoff WE, Cheng VCC, Chiotos K, et al. Coronavirus disease 2019 (COVID-19) research agenda for healthcare epidemiology.

Infect Control Hosp Epidemiol [Internet]. 2022;43(2):156–66. Available from: https://www.scopus.com/inward/record.uri?eid=2-s2.0-85124850650&doi=10.1017%2Fice.2021.25&partnerID=40&md5=4e1eff8b6f1d7f8da9e462f5af9087d7

23. Liu N, Chee ML, Niu C, Pek PP, Siddiqui FJ, Ansah JP, et al. Coronavirus disease 2019 (COVID-19): an evidence map of medical literature. BMC Med Res Methodol [Internet]. 2020;20(1):177. Available from: http://ovidsp.ovid.com/ovidweb.cgi?T=JS&PAGE=reference&D=med17&NEWS=N&AN=32615936

24. Dudovitz RN, Russ S, Berghaus M, Iruka IU, DiBari J, Foney DM, et al. COVID-19 and Children’s Well-Being: A Rapid Research Agenda. Matern Child Health J [Internet]. 2021;25(11):1655–69. Available from: https://www.scopus.com/inward/record.uri?eid=2-s2.0-85113325591&doi=10.1007%2Fs10995-021-03207-2&partnerID=40&md5=9ae76a064ffc2f51d08e0806fb841ba2

25. Rylett RJ, Alary F, Goldberg J, Rogers S, Versteegh P. Covid-19 and Priorities for Research in Aging. Canadian Journal on Aging [Internet]. 2020;39(4):500–5. Available from: https://www.scopus.com/inward/record.uri?eid=2-s2.0-85094821612&doi=10.1017%2FS0714980820000331&partnerID=40&md5=00c41786ef98411c2832d8edca52efc6

26. Lindsley WG, Blachère FM, Burton NC, Christensen B, Estill CF, Fisher EM, et al. COVID-19 and the workplace: Research questions for the aerosol science community. Aerosol Science and Technology [Internet]. 2020;54(10):1117–23. Available from: https://www.scopus.com/inward/record.uri?eid=2-s2.0-85088931860&doi=10.1080%2F02786826.2020.1796921&partnerID=40&md5=a4fc3c4695fd7ce1ecba1363b891828c

27. Bonney EY, Lamptey H, Puplampu P, Kyei GB. COVID-19 pandemic: Ten research questions Africa must answer for itself. Ghana Med J [Internet]. 2020;54(4):107–12. Available from: https://www.scopus.com/inward/record.uri?eid=2-s2.0-85101400846&doi=10.4314%2FGMJ.V54I4S.17&partnerID=40&md5=de459e2a23beef7754f2225fdd431708

28. Semenza JC, Adlhoch C, Baka A, Broberg E, Cenciarelli O, de Angelis S, et al. COVID-19 research priorities for non-pharmaceutical public health and social measures. Epidemiol Infect [Internet]. 2021; Available from: https://www.scopus.com/inward/record.uri?eid=2-s2.0-85103794609&doi=10.1017%2FS0950268821000716&partnerID=40&md5=29df0760ffe3e15f34f6231001c1e170

29. Shook LL, Fallah PN, Silberman JN, Edlow AG. COVID-19 Vaccination in Pregnancy and Lactation: Current Research and Gaps in Understanding. Front Cell Infect Microbiol [Internet]. 2021;11. Available from:

https://www.scopus.com/inward/record.uri?eid=2-s2.0-85116259784&doi=10.3389%2Ffcimb.2021.735394&partnerID=40&md5=805f34d2df3467181578c017a484cf0b

30. de la Rica R, Borges M, Gonzalez-Freire M. COVID-19: In the Eye of the Cytokine Storm. Front Immunol [Internet]. 2020;11. Available from: https://www.scopus.com/inward/record.uri?eid=2-s2.0-85092279971&doi=10.3389%2Ffimmu.2020.558898&partnerID=40&md5=e2bfc45f53934fff07413f5d27b237cd

31. Liu KY, Howard R, Banerjee S, Comas-Herrera A, Goddard J, Knapp M, et al. Dementia wellbeing and COVID-19: Review and expert consensus on current research and knowledge gaps. Int J Geriatr Psychiatry [Internet]. 2021;36(11):1597–639. Available from: https://www.scopus.com/inward/record.uri?eid=2-s2.0-85106582153&doi=10.1002%2Fgps.5567&partnerID=40&md5=01592383a8557814d5d32329dbd95309

32. Errett NA, Howarth M, Shoaf K, Couture M, Ramsey S, Rosselli R, et al. Developing an environmental health sciences covid-19 research agenda: Results from the NIEHS disaster research response (DR2) work group's modified delphi method. Int J Environ Res Public Health [Internet]. 2020;17(18):1–11. Available from: https://www.scopus.com/inward/record.uri?eid=2-s2.0-85091065368&doi=10.3390%2Fijerph17186842&partnerID=40&md5=4343ef9ca9a4094d40fbf3639e81b90f

33. Uyeki TM, Erlandson KJ, Korch G, O'Hara M, Wathen M, Hu-Primmer J, et al. Development of Medical Countermeasures to Middle East Respiratory Syndrome Coronavirus. Emerg Infect Dis [Internet]. 2016;22(7):E1–11. Available from: https://www.scopus.com/inward/record.uri?eid=2-s2.0-85017301680&doi=10.3201%2FEID2207.160022&partnerID=40&md5=8003f085d0c386e5fb3eeb64e7b0ed83

34. Payne DC, Franzke LH, Stehr-Green PA, Schwartz B, McNeil MM. Development of the Vaccine Analytic Unit's research agenda for investigating potential adverse events associated with anthrax vaccine adsorbed. Pharmacoepidemiol Drug Saf [Internet]. 2007;16(1):46–54. Available from: https://www.scopus.com/inward/record.uri?eid=2-s2.0-33846529454&doi=10.1002%2Fpds.1213&partnerID=40&md5=aaa2daf5b85894f157a75957f818ba9f

35. Poland GA, Kennedy RB, Ovsyannikova IG, Palacios R, Ho PL, Kalil J. Development of vaccines against Zika virus. Lancet Infect Dis [Internet]. 2018;18(7):e211–9. Available from: https://www.scopus.com/inward/record.uri?eid=2-s2.0-85041608916&doi=10.1016%2FS1473-3099%2818%2930063-X&partnerID=40&md5=9fd0c4601d5002cb96b2652ba0c59b07

36. The COVID-19 Research GloPID-R Synergies Meeting Working Group & Meeting Co-Chairs. Ending COVID-19: progress and gaps in research-highlights of the July 2020 GloPID-R COVID-19 Research Synergies Meetings. BMC Med [Internet]. 2020;18(1):342. Available from: https://www.scopus.com/inward/record.uri?eid=2-s2.0-85094860351&doi=10.1186%2Fs12916-020-01807-3&partnerID=40&md5=b8a7782283a39827c65a6dedbd20fe76

37. Cowling BJ, Leung GM. Epidemiological research priorities for public health control of the ongoing global novel coronavirus (2019-nCoV) outbreak. Eurosurveillance [Internet]. 2020;25(6). Available from: https://www.scopus.com/inward/record.uri?eid=2-s2.0-85080843714&doi=10.2807%2F1560-7917.ES.2020.25.6.2000110&partnerID=40&md5=99c7759781552347254a262deba52635

38. Evans K, Janiszewski H, Evans C, Spiby H. Establishing information needs and research priorities in response to the Covid-19 pandemic in the local maternity setting. Midwifery [Internet]. 2021;95. Available from: https://www.scopus.com/inward/record.uri?eid=2-s2.0-85099865897&doi=10.1016%2Fj.midw.2021.102922&partnerID=40&md5=0c2309256ef623b3ea5f354cb1906ad0

39. Clemens J. Evaluation of vaccines against enteric infections: A clinical and public health research agenda for developing countries. Philosophical Transactions of the Royal Society B: Biological Sciences [Internet]. 2011;366(1579):2799–805. Available from: https://www.scopus.com/inward/record.uri?eid=2-s2.0-80052483841&doi=10.1098%2Frstb.2011.0033&partnerID=40&md5=bcebc742f4ee7b469b51b26add42e11a

40. European Food Safety Authority. Event report: workshop on research gap analysis in animal influenza 8 and 9 January 2015, Parma [Internet]. 2015. Available from: http://www.efsa.europa.eu/sites/default/files/corporate_publications/files/787e.pdf

41. Hand MW, Alexander C, Lyman B, Parchment J, Joseph ML, Chipps E. Filling the Knowledge Gap for Nurse Leaders: Next Steps Following COVID-19. Nurse Lead [Internet]. 2021;19(6):616–21. Available from: https://www.scopus.com/inward/record.uri?eid=2-s2.0-85112859578&doi=10.1016%2Fj.mnl.2021.07.005&partnerID=40&md5=9292d44e0df7d8fffcf0be75cd574e92

42. Destoumieux-Garzón D, Matthies-Wiesler F, Bierne N, Binot A, Boissier J, Devouge A, et al. Getting out of crises: Environmental, social-ecological and evolutionary research is needed to avoid future risks of pandemics. Environ Int [Internet]. 2022;158. Available from: https://www.scopus.com/inward/record.uri?eid=2-s2.0-85116679915&doi=10.1016%2Fj.envint.2021.106915&partnerID=40&md5=34ca7176a92e1271f64e906fe0819686

43. Vearey J, de Gruchy T, Maple N. Global health (security), immigration governance and Covid-19 in South(ern) Africa: An evolving research agenda. J Migr Health [Internet]. 2021;3. Available from: https://www.scopus.com/inward/record.uri?eid=2-s2.0-85111539858&doi=10.1016%2Fj.jmh.2021.100040&partnerID=40&md5=af036ae6e77c88cd16788330d0373ed8

44. Abu-Raya B, Madhi SA, Omer SB, Amirthalingam G, Giles ML, Flanagan KL, et al. Global Perspectives on Immunization Against SARS-CoV-2 During Pregnancy and Priorities for Future Research: An International Consensus Paper From the World Association of Infectious Diseases and Immunological Disorders. Front Immunol [Internet]. 2021;12. Available from: https://www.scopus.com/inward/record.uri?eid=2-s2.0-85122478108&doi=10.3389%2Ffimmu.2021.808064&partnerID=40&md5=664c276ac756d0c9ee784df51939a405

45. Etti M, Alger J, Salas SP, Saggers R, Ramdin T, Endler M, et al. Global research priorities for COVID-19 in maternal, reproductive and child health: Results of an international survey. PLoS One [Internet]. 2021;16(9). Available from: https://www.scopus.com/inward/record.uri?eid=2-s2.0-85115811535&doi=10.1371%2Fjournal.pone.0257516&partnerID=40&md5=4dce59387cd56cc9b4fed622ed0c68af

46. Centers for Disease Control and Prevention (CDC). Guidelines for surveillance, prevention, and control of West Nile virus infection - United States. Morbidity and Mortality Weekly Report. 2000;49(2):25–8.

47. Baral P. Health Systems and Services During COVID-19: Lessons and Evidence From Previous Crises: A Rapid Scoping Review to Inform the United Nations Research Roadmap for the COVID-19 Recovery. International Journal of Health Services [Internet]. 2021;51(4):474–93. Available from: https://www.scopus.com/inward/record.uri?eid=2-s2.0-85102769945&doi=10.1177%2F0020731421997088&partnerID=40&md5=6faa88224b99af17d0be556ba25e4ef1

48. Simpson K, Heymann D, Brown CS, Edmunds WJ, Elsgaard J, Fine P, et al. Human monkeypox – After 40 years, an unintended consequence of smallpox eradication. Vaccine [Internet]. 2020;38(33):5077–81. Available from: https://www.scopus.com/inward/record.uri?eid=2-s2.0-85087469022&doi=10.1016%2Fj.vaccine.2020.04.062&partnerID=40&md5=175f753bde765e498638ccac8b104146

49. Vallès X, Stenseth NC, Demeure C, Horby P, Mead PS, Cabanillas O, et al. Human plague: An old scourge that needs new answers. PLoS Negl Trop Dis [Internet]. 2020;14(8):1–22. Available from: https://www.scopus.com/inward/record.uri?eid=2-s2.0-85090000025&doi=10.1371%2Fjournal.pntd.0008251&partnerID=40&md5=34aa1d29940131e56efbbe952d41e2a2

50. Siegfried AL, Carbone EG, Meit MB, Kennedy MJ, Yusuf H, Kahn EB. Identifying and Prioritizing Information Needs and Research Priorities of Public Health Emergency Preparedness and Response Practitioners. Disaster Med Public Health Prep [Internet]. 2017;11(5):552–61. Available from: https://www.scopus.com/inward/record.uri?eid=2-s2.0-85015881639&doi=10.1017%2Fdmp.2016.198&partnerID=40&md5=67f9c23260f1eb0a03d7111e21972815

51. Zhang H, Shaw R. Identifying research trends and gaps in the context of covid-19. Int J Environ Res Public Health [Internet]. 2020;17(10). Available from: https://www.scopus.com/inward/record.uri?eid=2-s2.0-85084417394&doi=10.3390%2Fijerph17103370&partnerID=40&md5=66ed976fce6c0c9d94cda4815b4c513c

52. McMahon M, Nadigel J, Thompson E, Glazier RH. Informing Canada's health system response to COVID-19: Priorities for health services and policy research. Healthcare Policy [Internet]. 2020;16(1):1–27. Available from: https://www.scopus.com/inward/record.uri?eid=2-s2.0-85089714325&doi=10.12927%2FHCPOL.2020.26249&partnerID=40&md5=0a300b383363816ccf417154f78737cc

53. Rojek A, Horby P, Dunning J. Insights from clinical research completed during the west Africa Ebola virus disease epidemic. Lancet Infect Dis [Internet]. 2017;17(9):e280–92. Available from: https://www.scopus.com/inward/record.uri?eid=2-s2.0-85018340356&doi=10.1016%2FS1473-3099%2817%2930234-7&partnerID=40&md5=a5dfa17906861b5a98332717c968173a

54. Corbel V, Fonseca DM, Weetman D, Pinto J, Achee NL, Chandre F, et al. International workshop on insecticide resistance in vectors of arboviruses, December 2016, Rio de Janeiro, Brazil. Parasit Vectors [Internet]. 2017;10(1). Available from: https://www.scopus.com/inward/record.uri?eid=2-s2.0-85019999568&doi=10.1186%2Fs13071-017-2224-3&partnerID=40&md5=b7a2bc6ea098a50de4b696fe0ae397cf

55. Noel GJ, Davis JM, Ramilo O, Bradley JS, Connor E. Key clinical research priorities for the pediatric community during the COVID-19 pandemic. Pediatr Res [Internet]. 2021;89(4):730–2. Available from: https://www.scopus.com/inward/record.uri?eid=2-s2.0-85084729605&doi=10.1038%2Fs41390-020-0962-y&partnerID=40&md5=d318c1ebf79b19a7fc1a47350861110b

56. Yazdizadeh B, Ehsani-Chimeh E, Zendehdel K, Mobinizadeh M, Mesgarpour B, Fakoorfard Z. Knowledge gaps and national research priorities for COVID-19 in Iran. Health Res Policy Syst [Internet]. 2022;20(1). Available from: https://www.scopus.com/inward/record.uri?eid=2-s2.0-

85125613370&doi=10.1186%2Fs12961-021-00805-y&partnerID=40&md5=f51f53a6a9d6b20c14224fe6ba3dec95

57. Jacobsen KH, Alonso Aguirre A, Bailey CL, Baranova A V, Crooks AT, Croitoru A, et al. Lessons from the ebola outbreak: Action items for emerging infectious disease preparedness and response. Ecohealth [Internet]. 2016;13(1):200–12. Available from: https://www.scopus.com/inward/record.uri?eid=2-s2.0-84975709273&doi=10.1007%2Fs10393-016-1100-5&partnerID=40&md5=00e441cd27bc351b3c9b7a968f0435ca

58. Sutton J, Rivera Y, Sell TK, Moran MB, Bennett Gayle D, Schoch-Spana M, et al. Longitudinal Risk Communication: A Research Agenda for Communicating in a Pandemic. Health Secur [Internet]. 2021;19(4):370–8. Available from: https://www.scopus.com/inward/record.uri?eid=2-s2.0-85108238755&doi=10.1089%2Fhs.2020.0161&partnerID=40&md5=2c1b7432b187cd37566575eb31435144

59. Dao TL, To MM, Nguyen TD, Hoang VT. Mapping COVID-19 related research from Vietnam: a scoping review. J Prev Med Hyg [Internet]. 2022;63(1):E166–73. Available from: https://www.scopus.com/inward/record.uri?eid=2-s2.0-85131222708&doi=10.15167%2F2421-4248%2Fjpmh2022.63.1.1720&partnerID=40&md5=9541c837cd6467942d410cbf6c50d9cf

60. Grace D, Mutua F, Ochungo P, Kruska R, Jones K, Brierley L, et al. Mapping of poverty and likely zoonoses hotspots. Zoonoses Project 4 [Internet]. 2012; Available from: http://r4d.dfid.gov.uk/pdf/outputs/livestock/ZooMapDFIDreport18June2012FINALsm.pdf

61. Aguanno R, ElIdrissi A, Elkholy AA, Ben Embarek P, Gardner E, Grant R, et al. MERS: Progress on the global response, remaining challenges and the way forward. Antiviral Res [Internet]. 2018;159:35–44. Available from: https://www.scopus.com/inward/record.uri?eid=2-s2.0-85053772456&doi=10.1016%2Fj.antiviral.2018.09.002&partnerID=40&md5=5922702cc41b193cd674b86a250fc91e

62. Memish ZA, Perlman S, Van Kerkhove MD, Zumla A. Middle East respiratory syndrome. The Lancet [Internet]. 2020;395(10229):1063–77. Available from: https://www.scopus.com/inward/record.uri?eid=2-s2.0-85082185623&doi=10.1016%2FS0140-6736%2819%2933221-0&partnerID=40&md5=ac4be5b274781663b60c32171dcdd9fc

63. Malik A, Elkholy AA, Khan W, Hassounah S, Abubakar A, Tran Minh N, et al. Middle East respiratory syndrome coronavirus: current knowledge and future considerations [Internet]. Vol. 22. 2016. 533–542 p. Available from: https://pesquisa.bvsalud.org/gim/resource/en/emr-181510

64. Shrivastava SR, Shrivastava PS. Necessity to Prioritize Research Activities for a Better Public Health Response to Coronavirus Disease-2019 Pandemic. Adv Biomed Res [Internet]. 2020;9:34. Available from: http://ovidsp.ovid.com/ovidweb.cgi?T=JS&PAGE=reference&D=pmnm5&NEWS=N&AN=33072646

65. Khan SH, Goba A, Chu M, Roth C, Healing T, Marx A, et al. New opportunities for field research on the pathogenesis and treatment of Lassa fever. Antiviral Res [Internet]. 2008;78(1):103–15. Available from: https://www.scopus.com/inward/record.uri?eid=2-s2.0-40749093780&doi=10.1016%2Fj.antiviral.2007.11.003&partnerID=40&md5=cb075574e10ba7b9810a1c9e5fc58629

66. Weerakkody S, Arina P, Glenister J, Cottrell S, Boscaini-Gilroy G, Singer M, et al. Non-invasive respiratory support in the management of acute COVID-19 pneumonia: considerations for clinical practice and priorities for research. Lancet Respir Med [Internet]. 2022;10(2):199–213. Available from: https://www.scopus.com/inward/record.uri?eid=2-s2.0-85123850320&doi=10.1016%2FS2213-2600%2821%2900414-8&partnerID=40&md5=b13fa02e1592130d304171a8fa9415c9

67. Gupta S, Starr MK, Farahani RZ, Asgari N. Pandemics/Epidemics: Challenges and Opportunities for Operations Management Research. Manufacturing and Service Operations Management [Internet]. 2022;24(1):1–23. Available from: https://www.scopus.com/inward/record.uri?eid=2-s2.0-85124487334&doi=10.1287%2FMSOM.2021.0965&partnerID=40&md5=3f1d56adc4dbcbbf2b4434237bdeb28f

68. Faghy MA, Arena R, Babu AS, Christle JW, Marzolini S, Popovic D, et al. Post pandemic research priorities: A consensus statement from the HL-PIVOT. Prog Cardiovasc Dis [Internet]. 2022;73:2–16. Available from: https://www.scopus.com/inward/record.uri?eid=2-s2.0-85134722128&doi=10.1016%2Fj.pcad.2022.07.001&partnerID=40&md5=66324efb32fd14f3b166645cb6561070

69. Lentine KL, Mannon RB, Josephson MA. Practicing With Uncertainty: Kidney Transplantation During the COVID-19 Pandemic. American Journal of Kidney Diseases [Internet]. 2021;77(5):777–85. Available from: https://www.ncbi.nlm.nih.gov/pmc/articles/PMC7946342/pdf/main.pdf

70. Norton AJ, Wiysonge CS, Habarugira JMV, White NJ, Tufet Bayona M, Hagen HE, et al. Priorities for COVID-19 research response and preparedness in low-resource settings. The Lancet [Internet]. 2021 May 22 [cited 2022 Jun 6];397(10288):1866–8. Available from: http://www.thelancet.com/article/S0140673621009806/fulltext

71. Manning JC, Bramley L, Coad J, Evans C, Evans K, Tinkler L, et al. Priorities for research during the Coronavirus SARS-CoV-2 (COVID-19) pandemic and beyond: a survey of

nurses, midwives and health visitors in the United Kingdom. Journal of Research in Nursing [Internet]. 2021;26(5):442–54. Available from: https://www.scopus.com/inward/record.uri?eid=2-s2.0-85112134439&doi=10.1177%2F17449871211018737&partnerID=40&md5=1c829d4f034d137adef5964df6aeca10

72. Ault KA, Heine RP, Riley LE. Programmatic and research priorities for improving influenza immunization of pregnant women. Am J Obstet Gynecol [Internet]. 2012;207(3):S75-7. Available from: http://ovidsp.ovid.com/ovidweb.cgi?T=JS&PAGE=reference&D=med9&NEWS=N&AN=22920064

73. Cristina Cassetti M, Pierson TC, Jean Patterson L, Bok K, DeRocco AJ, Deschamps AM, et al. Prototype Pathogen Approach for Vaccine and Monoclonal Antibody Development: A Critical Component of the NIAID Plan for Pandemic Preparedness. J Infect Dis [Internet]. 2022; Available from: http://ovidsp.ovid.com/ovidweb.cgi?T=JS&PAGE=reference&D=medp&NEWS=N&AN=35876700

74. Azim T, Bhushan A, Del Rio Vilas VJ, Srivastava R, Wijesinghe PR, Ofrin R, et al. Public health research priorities for WHO on COVID-19 in the South-East Asia Region: results of a prioritization survey. Health Res Policy Syst [Internet]. 2022;20(1). Available from: https://www.scopus.com/inward/record.uri?eid=2-s2.0-85137186625&doi=10.1186%2Fs12961-022-00862-x&partnerID=40&md5=e757314f795df9309ee1371822fc3689

75. Highet M. Rapid Scoping Review on the Topic of Ensuring Social Protection and Basic Services to Inform the United Nations Framework for the Immediate Socioeconomic Response to COVID-19. International Journal of Health Services [Internet]. 2021;51(4):462–73. Available from: https://www.scopus.com/inward/record.uri?eid=2-s2.0-85102742738&doi=10.1177%2F00207314211024896&partnerID=40&md5=9cff14353bff0f36e080987d74538d82

76. Papa A, Mirazimi A, Köksal I, Estrada-Pena A, Feldmann H. Recent advances in research on Crimean-Congo hemorrhagic fever. Journal of Clinical Virology [Internet]. 2015;64:137–43. Available from: https://www.scopus.com/inward/record.uri?eid=2-s2.0-84923553145&doi=10.1016%2Fj.jcv.2014.08.029&partnerID=40&md5=b8c98267b7d283510a8e98ded9b7324f

77. Yassi A, Moore D, Fitzgerald JM, Bigelow P, Hon CY, Bryce E. Research gaps in protecting healthcare workers from SARS and other respiratory pathogens: An interdisciplinary, multi-stakeholder, evidence-based approach. J Occup Environ Med [Internet]. 2005;47(1):41–50. Available from: http://ovidsp.ovid.com/ovidweb.cgi?T=JS&PAGE=reference&D=emed9&NEWS=N&AN=40129012

78. Folayan MO, Haire B, Allman D, Yakubu A, Afolabi MO. Research priorities during infectious disease emergencies in West Africa. BMC Res Notes [Internet]. 2018;11(1). Available from: https://www.scopus.com/inward/record.uri?eid=2-s2.0-85042771792&doi=10.1186%2Fs13104-018-3263-3&partnerID=40&md5=df5d2bcc27bd40d927b35ad3865503dc

79. Simpson G, Quesada F, Chatterjee P, Kakkar M, Chersich MF, Thys S. Research priorities for control of zoonoses in South Africa. Trans R Soc Trop Med Hyg [Internet]. 2021;115(5):538–50. Available from: https://www.scopus.com/inward/record.uri?eid=2-s2.0-85106538747&doi=10.1093%2Ftrstmh%2Ftrab039&partnerID=40&md5=38e53cf10e44fb634d073a0f414c5581

80. Tong A, Sorrell TC, Black AJ, Caillaud C, Chrzanowski W, Li E, et al. Research priorities for COVID-19 sensor technology. Nat Biotechnol [Internet]. 2021;39(2):144–7. Available from: https://www.scopus.com/inward/record.uri?eid=2-s2.0-85100204797&doi=10.1038%2Fs41587-021-00816-8&partnerID=40&md5=99faff8b5216b4ef6dd056ee913b0fef

81. Research priorities for diarrhoeal disease vaccines: Memorandum from a WHO meeting. Bull World Health Organ [Internet]. 1991;69(6):667–76. Available from: https://www.scopus.com/inward/record.uri?eid=2-s2.0-0026334839&partnerID=40&md5=14974dded9e44f0a0a5ef719dd1da702

82. Wiwanitkit V, Shi B, Xia S, Yang GJ, Zhou XN, Liu J. Research priorities in modeling the transmission risks of H7N9 bird flu. Infect Dis Poverty [Internet]. 2013;2(1). Available from: https://www.scopus.com/inward/record.uri?eid=2-s2.0-84899798336&doi=10.1186%2F2049-9957-2-17&partnerID=40&md5=c9bbc867549bdeb03dae82a041900b58

83. French MB, Loeb MB, Richardson C, Singh B. Research preparedness paves the way to respond to pandemic H1N1 2009 influenza virus. Canadian Journal of Infectious Diseases and Medical Microbiology [Internet]. 2009;20(3):e63–6. Available from: https://www.scopus.com/inward/record.uri?eid=2-s2.0-70350169655&doi=10.1155%2F2009%2F798387&partnerID=40&md5=0e7f9aef6a48fe2869d13a078ca8f1d6

84. Board on Health Sciences P, Board on Global H, Board on Population H, Public Health P, Board on Life S, Institute of M, et al. Research Priorities to Inform Public Health and Medical Practice for Ebola Virus Disease: Workshop in Brief. 2014; Available from: http://ovidsp.ovid.com/ovidweb.cgi?T=JS&PAGE=reference&D=medp&NEWS=N&AN=26020089

85. Polašek O, Wazny K, Adeloye D, Song P, Chan KY, Bojude DA, et al. Research priorities to reduce the impact of COVID-19 in low- and middle-income countries. J Glob Health [Internet]. 2022;12:1–14. Available from:

https://www.scopus.com/inward/record.uri?eid=2-s2.0-85128946942&doi=10.7189%2Fjogh.12.09003&partnerID=40&md5=158b62148b30e91cea02a95260b5a229

86. Niederman MS, Richeldi L, Chotirmall SH, Bai C. Rising to the challenge of COVID-19: Advice for pulmonary and critical care and an agenda for research. Am J Respir Crit Care Med [Internet]. 2020;201(9):1019–22. Available from: https://www.scopus.com/inward/record.uri?eid=2-s2.0-85083212823&doi=10.1164%2FRCCM.202003-0741ED&partnerID=40&md5=eace056e4f729d1743cc8cc40c4b986f

87. Reese G, Hamann KRS, Heidbreder LM, Loy LS, Menzel C, Neubert S, et al. SARS-Cov-2 and environmental protection: A collective psychology agenda for environmental psychology research. J Environ Psychol [Internet]. 2020;70. Available from: https://www.scopus.com/inward/record.uri?eid=2-s2.0-85086388519&doi=10.1016%2Fj.jenvp.2020.101444&partnerID=40&md5=4a17e1494f54d9e9b491df1e77d13667

88. Eder M, Cortes F, Teixeira de Siqueira Filha N, Araújo de França G V, Degroote S, Braga C, et al. Scoping review on vector-borne diseases in urban areas: Transmission dynamics, vectorial capacity and co-infection. Infect Dis Poverty [Internet]. 2018;7(1). Available from: https://www.scopus.com/inward/record.uri?eid=2-s2.0-85052625339&doi=10.1186%2Fs40249-018-0475-7&partnerID=40&md5=e34fa3ccc5bbc966a45184cba518b71e

89. Mehta K, Zodpey S, Banerjee P, Pocius SL, Dhaliwal BK, DeLuca A, et al. Shifting research priorities in maternal and child health in the COVID-19 pandemic era in India: A renewed focus on systems strengthening. PLoS One [Internet]. 2021;16(8). Available from: https://www.scopus.com/inward/record.uri?eid=2-s2.0-85112774087&doi=10.1371%2Fjournal.pone.0256099&partnerID=40&md5=754405778eadc9a8953a293ec5a6dac6

90. Hult Khazaie D, Khan SS. Social psychology and pandemics: Exploring consensus about research priorities and strategies using the Delphi method. Asian J Soc Psychol [Internet]. 2020;23(4):363–71. Available from: https://www.scopus.com/inward/record.uri?eid=2-s2.0-85096008358&doi=10.1111%2Fajsp.12442&partnerID=40&md5=5c9b42cff27bafbca508b5d5c7d11293

91. Otmani del Barrio M, Simard F, Caprara A. Supporting and strengthening research on urban health interventions for the prevention and control of vector-borne and other infectious diseases of poverty: Scoping reviews and research gap analysis. Infect Dis Poverty [Internet]. 2018;7(1). Available from: https://idpjournal.biomedcentral.com/counter/pdf/10.1186/s40249-018-0462-z.pdf

92. Milone M, Carrano FM, Letić E, Shamiyeh A, Forgione A, Eom BW, et al. Surgical challenges and research priorities in the era of the COVID-19 pandemic: EAES

membership survey. Surg Endosc [Internet]. 2020;34(10):4225–32. Available from: https://www.scopus.com/inward/record.uri?eid=2-s2.0-85088986710&doi=10.1007%2Fs00464-020-07835-7&partnerID=40&md5=e0e26a74fe714e04b389bb0e03805210

93. Johnson KM. The arenaviruses: some priorities for future research. Bull World Health Organ [Internet]. 1975;52(4):761–3. Available from: https://www.scopus.com/inward/record.uri?eid=2-s2.0-0016647452&partnerID=40&md5=3b39d1c614686b145855cc73d508a544

94. Rizzoli A, Jiménez-Clavero MA, Barzon L, Cordioli P, Figuerola J, Koraka P, et al. The challenge of west nile virus in Europe: Knowledge gaps and research priorities. Eurosurveillance [Internet]. 2015;20(20). Available from: https://www.scopus.com/inward/record.uri?eid=2-s2.0-84930403033&doi=10.2807%2F1560-7917.ES2015.20.20.21135&partnerID=40&md5=094ab05e627870d6a7ea28b4204e9f67

95. Barouki R, Kogevinas M, Audouze K, Belesova K, Bergman A, Birnbaum L, et al. The COVID-19 pandemic and global environmental change: Emerging research needs. Environ Int [Internet]. 2021;146. Available from: https://www.scopus.com/inward/record.uri?eid=2-s2.0-85096680168&doi=10.1016%2Fj.envint.2020.106272&partnerID=40&md5=a5d2d8b7c0e80e544e4dfc63c08508d0

96. Sharp GC, Fraser A, Sawyer G, Kountourides G, Easey KE, Ford G, et al. The COVID-19 pandemic and the menstrual cycle: Research gaps and opportunities. Int J Epidemiol [Internet]. 2022;51(3):691–700. Available from: https://www.scopus.com/inward/record.uri?eid=2-s2.0-85131902566&doi=10.1093%2Fije%2Fdyab239&partnerID=40&md5=49739482d5c2e45e8aeacfb51c59e3b2

97. Agarwal A, Chaurasia D. The expanding arms of Zika virus: An updated review with recent Indian outbreaks. Rev Med Virol [Internet]. 2021;31(1):1–9. Available from: https://onlinelibrary.wiley.com/doi/10.1002/rmv.2145

98. Blasiak A, Truong ATL, Remus A, Hooi L, Seah SGK, Wang P, et al. The IDentif.AI-x pandemic readiness platform: Rapid prioritization of optimized COVID-19 combination therapy regimens. NPJ Digit Med [Internet]. 2022;5(1). Available from: https://www.scopus.com/inward/record.uri?eid=2-s2.0-85133127917&doi=10.1038%2Fs41746-022-00627-4&partnerID=40&md5=f6a951c3666f70e6990b658e5007d21b

99. Gupta N, Singhai M, Garg S, Shah D, Sood V, Singh SK. The missing pieces in the jigsaw and need for cohesive research amidst coronavirus infectious disease 2019 global response. Med J Armed Forces India [Internet]. 2020;76(2):132–5. Available from: https://www.ncbi.nlm.nih.gov/pmc/articles/PMC7141472/pdf/main.pdf

100. Norton A, De La Horra Gozalo A, Feune de Colombi N, Alobo M, Mutheu Asego J, Al-Rawni Z, et al. The remaining unknowns: a mixed methods study of the current and global health research priorities for COVID-19. BMJ Glob Health [Internet]. 2020 Jul 29 [cited 2020 Aug 18];5(7):e003306. Available from: http://gh.bmj.com/lookup/doi/10.1136/bmjgh-2020-003306

101. Calnan M, Gadsby EW, Kondé MK, Diallo A, Rossman JS. The response to and impact of the ebola epidemic: Towards an agenda for interdisciplinary research. Int J Health Policy Manag [Internet]. 2018;7(5):402–11. Available from: https://www.scopus.com/inward/record.uri?eid=2-s2.0-85049975106&doi=10.15171%2Fijhpm.2017.104&partnerID=40&md5=fc7979eb894a2157165779bbef890449

102. Jarman MP, Bergmark RW, Chhabra K, Scott JW, Shrime M, Cooper Z, et al. The surgical health services research agenda for the COVID-19 pandemic. Ann Surg [Internet]. 2020;272(3):e226–9. Available from: https://journals.lww.com/annalsofsurgery/Fulltext/2020/09000/The_Surgical_Health_Services_Research_Agenda_for.50.aspx

103. Coopersmith CM, Antonelli M, Bauer SR, Deutschman CS, Evans LE, Ferrer R, et al. The Surviving Sepsis Campaign: Research Priorities for Coronavirus Disease 2019 in Critical Illness. Crit Care Med [Internet]. 2021;598–622. Available from: https://www.scopus.com/inward/record.uri?eid=2-s2.0-85103228622&doi=10.1097%2FCCM.0000000000004895&partnerID=40&md5=43553f8f8eda181b94789f13eb48d2b7

104. Benowitz NL, Goniewicz ML, Halpern-Felsher B, Krishnan-Sarin S, Ling PM, O’Connor RJ, et al. Tobacco product use and the risks of SARS-CoV-2 infection and COVID-19: current understanding and recommendations for future research. Lancet Respir Med [Internet]. 2022;10(9):900–15. Available from: https://www.scopus.com/inward/record.uri?eid=2-s2.0-85137104739&doi=10.1016%2FS2213-2600%2822%2900182-5&partnerID=40&md5=554674945e5a22eb46bce213e6aa8e08

105. Sinha M, Kumar M, Zeitz L, Collins PY, Kumar S, Fisher S, et al. Towards mental health friendly cities during and after COVID-19. Cities Health [Internet]. 2021;5:S89–92. Available from: https://www.scopus.com/inward/record.uri?eid=2-s2.0-85131597503&doi=10.1080%2F23748834.2020.1790251&partnerID=40&md5=c16e7d0c96c23de2b036ae3ed7508b12

106. Bausch DG, Sprecher AG, Jeffs B, Boumandouki P. Treatment of Marburg and Ebola hemorrhagic fevers: A strategy for testing new drugs and vaccines under outbreak conditions. Antiviral Res [Internet]. 2008;78(1):150–61. Available from: https://www.scopus.com/inward/record.uri?eid=2-s2.0-40749123737&doi=10.1016%2Fj.antiviral.2008.01.152&partnerID=40&md5=3d7985932aeaae9ee8a031f4590a8d0b

107. Stegenga J. Viruses without borders and the medical research agenda. Int J Risk Saf Med [Internet]. 2022;33(3):217–21. Available from: https://www.scopus.com/inward/record.uri?eid=2-s2.0-85137008108&doi=10.3233%2FJRS-227016&partnerID=40&md5=48866474bbb5eb6d39e3b6f7cb60451e

108. Wikan N, Smith DR. Zika virus from a Southeast Asian perspective. Asian Pac J Trop Med [Internet]. 2017;10(1):1–5. Available from: https://www.scopus.com/inward/record.uri?eid=2-s2.0-85009727807&doi=10.1016%2Fj.apjtm.2016.11.013&partnerID=40&md5=e1c440db78d007dc52d23d9a1a39fe12

109. Wilder-Smith A, Gubler DJ, Weaver SC, Monath TP, Heymann DL, Scott TW. Epidemic arboviral diseases: priorities for research and public health. Lancet Infect Dis [Internet]. 2017;17(3):e101–6. Available from: https://www.scopus.com/inward/record.uri?eid=2-s2.0-85008210285&doi=10.1016%2FS1473-3099%2816%2930518-7&partnerID=40&md5=dd546ae58ca43dbe0f23a14ff12eeeb1

110. Cowling BJ, Yu H. Ebola: Worldwide dissemination risk and response priorities. The Lancet [Internet]. 2015 Jan 3 [cited 2024 Feb 18];385(9962):7–9. Available from: http://www.thelancet.com/article/S014067361461895X/fulltext

111. Abela-Ridder B, Daszak P, Dauphin G, Donis R, Delarocque S, Formenty P, et al. Influenza and other emerging zoonotic diseases at the human-animal interphase, Joint Scientific Consultation [Internet]. 2010 [cited 2024 Feb 18]. Available from: https://openknowledge.fao.org/server/api/core/bitstreams/0c0b5faa-d72f-4f58-830c-33b35fddb4c4/content

112. Smith RD. Responding to global infectious disease outbreaks: Lessons from SARS on the role of risk perception, communication and management. Soc Sci Med [Internet]. 2006;63(12):3113–23. Available from: https://www.scopus.com/inward/record.uri?eid=2-s2.0-33750492952&doi=10.1016%2Fj.socscimed.2006.08.004&partnerID=40&md5=ff237f112ed819cdabce31d1d32969ea

113. World Health Organisation. A Coordinated Global Research Roadmap: 2019 Novel Coronavirus [Internet]. 2020. Available from: https://www.who.int/publications/m/item/a-coordinated-global-research-roadmap

114. African Academy of Sciences. Research and Development goals for COVID-19 in Africa. The African Academy of Sciences Priority Setting Exercise [Internet]. Available from: https://www.aasciences.africa/sites/default/files/2020-04/Research and Development Goals for COVID-19 in Africa.pdf

115. Africa Centres for Disease Control and Prevention (Africa CDC). Policy Paper: Research and Development Priorities for COVID-19 in Africa – Africa CDC [Internet]. 2021 [cited 2022 May 22]. Available from: https://africacdc.org/download/policy-paper-research-and-development-priorities-for-covid-19-in-africa/

116. United Nations. United Nations Research Roadmap for the COVID-19 Recovery [Internet]. 2020 [cited 2022 Jun 4]. Available from: https://www.un.org/en/coronavirus/communication-resources/un-research-roadmap-covid-19-recovery

117. Carson G. Research priorities for Long Covid: refined through an international multi-stakeholder forum. BMC Med [Internet]. 2021 [cited 2022 Mar 25]; Available from: https://doi.org/10.1186/s12916-021-01947-0

118. Paparella G, Norton A, Matulevics R, Jamieson N, Michelen M, Carson G. Recommendations and considerations for GloPID-R [Internet]. 2021 [cited 2022 Nov 2]. Available from: https://www.glopid-r.org/wp-content/uploads/2021/09/glopid-r-sag-report.pdf

119. Clarke E, Eriksson E, Kiura P, Dia A, Carrillo S. Creating a Publicly-Led Long Covid Research Agenda A mixed methods approach from Kenya and Malawi [Internet]. 2021 [cited 2022 Jun 1]. Available from: https://cms.wellcome.org/sites/default/files/2022-01/wellcome_long_covid_report.pdf

120. World Health Organization. How global research can end this pandemic and tackle future ones [Internet]. 2022 [cited 2022 Jun 1]. Available from: https://cdn.who.int/media/docs/default-source/blue-print/final-report-of-the-global-research-and-innovation-forum-2022.pdf?sfvrsn=4a59021f_5&download=true

121. US Department of Health and Human Services. National Research Action Plan [Internet]. 2022 [cited 2023 Jan 3]. Available from: https://www.covid.gov/assets/files/National-Research-Action-Plan-on-Long-COVID-08012022.pdf

122. Pan American Health Organization (PAHO). PAHO/WHO Regional research agenda related to Zika virus infection. Development of a research agenda for characterizing the Zika outbreak and its public health implications in the Americas. 2016.

123. World Health Organization (WHO). Monkeypox Strategic Preparedness, Readiness and Response Plan. 2022.

124. World Health Organization (WHO). Community-centred approaches to health emergencies: progress, gaps and research priorities-summary [Internet]. 2020. Available from: https://www.who.int/hac/techguidance/preparedness/health-emergency-and-disaster-risk-management-framework-

125. World Health Organization (WHO). Sexual transmission of Zika Virus: Current status, challenges and research priorities [Internet]. 2017 [cited 2024 Feb 18]. Available from: https://iris.who.int/bitstream/handle/10665/259583/WHO-RHR-17.23-eng.pdf?sequence=1
